# Global human myeloid replacement with peripheral progenitors induces interferonopathy and neurodegeneration

**DOI:** 10.21203/rs.3.rs-7762822/v1

**Published:** 2025-10-03

**Authors:** Jing Wang, Anna Warden, Bing Xia, Katie E Mostoller, Benjamin Brandon, Michelle Du, Nathanael Spann, Rick Z Li, Mana M Parast, Karen Mestan, Fatir Qureshi, Olivia Corradin, Christopher Glass, Nicole G Coufal

**Affiliations:** University of California, San Diego; University of California, San Diego; University of California, San Diego; University of California, San Diego; University of California, San Diego; University of California, San Diego; University of California; University of California, San Diego; University of California, San Diego; University of California, San Diego; Yale School of Medicine; Whitehead Institute for Biomedical; University of California, San Diego; Department of Pediatrics, University of California, San Diego, La Jolla, CA 92093, US

## Abstract

Microglia, the brain’s resident macrophages, arise from yolk sac hematopoietic progenitor cells (HPCs) that migrate into the brain during early embryonic development and differentiate in response to microenvironment-specific signals. The resulting spatial and stage-specific programs of gene expression enable microglia to function as key modulators of diverse homeostatic processes that include synaptic pruning, myelination, and neurogenesis throughout the lifespan. Dysregulation of these core microglia functions has been linked to numerous neurodevelopmental and neurodegenerative diseases. Although normally a closed niche, studies in mice indicate that peripheral monocytes, originating from hematopoietic stem cells (HSCs), can infiltrate the brain in circumstances in which the blood brain barrier is disrupted, with context-dependent protective or detrimental consequences. A major unanswered question with significant implications for therapy of CNS diseases driven by microglia dysfunction is the extent to which human HSC-derived cells can adopt microglia-like phenotypes that would allow them to restore brain homeostasis by replacement of pathologic HPC-derived microglia. To address this question, we directly compared the differentiation potential of primary human microglia, human iPSC-derived HPCs and human HSCs in the brain utilizing a murine xenotransplantation model. HSCs and monocytes were capable of differentiating into microglia like cells in this model, they also acquired a strong interferon, phagocytic, and antigen presenting phenotype distinct from engrafted primary human microglia and HPC-derived cells. Analyses of the epigenetic landscapes of the engrafted HPC and HSC-derived cells enabled identification of the transcription factors networks underlying ontogeny-specific brain myeloid fates. Ultimately, human peripheral myeloid cells in the CNS led to astrogliosis, myelin fragmentation and synaptic loss. These findings reveal transcriptional network differences influenced by ontogeny, and together with the accompanying study by Davtvan and colleagues provide critical insights for developing human microglial or bone marrow transplant-based therapies for CNS disorders.

## Introduction

Microglia, the resident innate immune cells of the central nervous system (CNS), play critical roles in maintaining brain homeostasis and supporting neuronal health from early development throughout the lifespan^1,2^. Microglia perform a variety of essential functions, including modulating astrocyte activation, promoting oligodendrocyte differentiation and myelination, and most notably, facilitating neuronal pruning in response to neural activity^3–5^. Microglial dystrophy or dysfunction is increasingly recognized as pathogenic in diverse neurological diseases ranging from lysosomal storage diseases to Alzheimer’s^6,7^. Brain myeloid cell replacement therefore hold promise for the treatment of neurological diseases by targeting the elimination of toxic microglia and introducing safe, functional myeloid replacements into the diseased brain^6–8^.

A key question in considering myeloid replacement therapies for neurological disease is the degree to which myeloid ontogeny influences innate immune function and ultimately brain health. Microglia possess a distinct yolk sac erythro-myeloid ontogeny^9–11^ that sets them apart from other tissue macrophages that arise from hematopoietic stem cells (HSCs). HSCs migrate through the fetal liver, the main site of prenatal hematopoiesis, before colonizing tissues^12–15^, and bone marrow HSCs give rise to blood monocytes to replenish macrophage populations throughout life^16^. The CNS is distinct in that it is protected by the endothelial (blood-brain barrier) and epithelial (choroid plexus) barriers^17,18^, limiting the entry of circulating monocytes and thus creating a closed homeostatic microenvironment for resident microglia. Human microglia exhibit a distinct transcriptional and epigenetic landscape tailored to and dependent upon the brain environment for their tissue resident identity^19,20^. Whether other human myeloid cell types can adapt and respond to the brain environment as precisely as microglia is poorly understood.

In the setting of intrinsic or extrinsic injury, an influx of bone marrow-derived macrophages from the circulation can occur, but whether this is protective or hastens neurodegeneration, worsening brain injury, is unclear and may be disease or context dependent^21,22^. Most studies to date have employed murine models, demonstrating that transplanted HSC-derived cells take on all but a small core of microglial transcriptional profiles, retaining some distinct morphological features^23^, differential responses to inflammatory stimuli^23^, and altered ATP-sensing abilities and intracellular calcium dynamics^24^. Furthermore, these cells show changes in phagocytic activity^25^ and exhibit a divergent transcriptomic profile compared to native microglia^26,27^. Overall, these murine studies have found that, although the myeloid ontogenies are distinct, the functional differences are modest and numerous studies have suggested monocytes, peripheral myeloid progenitors, or bone marrow transplant with enhanced microglial depletion as therapeutic avenues based on these murine studies^6,7,28,29^.

Thus far, questions about the ability of human myeloid progenitors and monocytes to recapitulate the human microglial cell fate remain unresolved. We sought to identify the degree to which ontogeny and brain environment regulate the human brain myeloid cell fate, and to identify candidate regulatory mechanisms underlying these processes. To address these questions, we applied a humanized murine model that genetically lacks microglia^30^, transplanting diverse human myeloid cell types used in bone marrow transplant approaches. We identified that xenotransplanted bone marrow progenitors exhibit distinct morphology, heterogeneity, and transcriptional and epigenetic signatures from primary or stem cell derived microglia—particularly in antigen presentation and interferon pathway activation—shaped by their developmental origin. Moreover, human myeloid cells displayed gene expression patterns distinct from those of mouse myeloid cells after CNS engraftment, indicating species-specific responses to the brain environment. These findings paired with the accompanying manuscript of Davtvan and Chadarevian et al. demonstrate that human myeloid ontogeny fundamentally determines the differentiation potential of myeloid cells within the brain environment and may negatively impact therapeutic full brain myeloid replacement strategies.

## Results

### Xenotransplantation to model myeloid ontogeny and environmental influence

**Figure 1:**
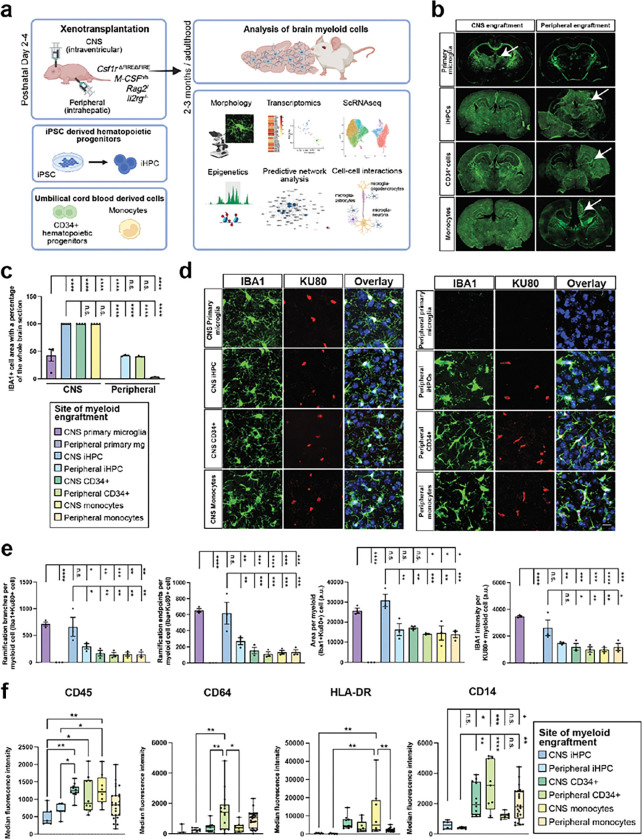
Xenotransplantation of diverse human myeloid lineages identifies variable CNS myeloid differentiation potential **(a) Schematic of the experimental design.** Induced pluripotent stem cell (iPSC)-derived induced hematopoietic progenitor cells (iHPCs), cord blood-derived CD34^+^ hematopoietic progenitors, and monocytes are xenotransplanted into the central nervous system (CNS, intraventricular) or peripherally (intrahepatic) of hFIRE (Csf1r^ΔFIRE/ΔFIRE^ Csf1^h/h^ Rag2^−/−^ Il2rg^−/−^) neonatal mice and assess by diverse means in adult mice. **(b) Immunofluorescent detection of transplanted cells** at the striatum and hippocampus levels, regions containing IBA1-positive cells (green) are indicated with white arrows. Scale bar = 250 μm. **(c)** Quantification of the brain area occupied by IBA1-positive myeloid cells based on CNS and peripheral engraftment. **(d)** Immunofluorescence of xenotransplanted human myeloid cells in the cortex, either engrafted in the CNS (left) or peripherally (right), labeled with IBA1 (myeloid marker) and Ku80 (human nuclear marker). Scale bar = 40 μm. **(e)** Quantification of myeloid cell ramification complexity, measured as the number of skeletonized branches (left) and endpoints (middle left) per cell, together with cell area (middle right) and IBA1^+^ signal intensity (right) **(f)** Flow cytometry analysis of brain myeloid cells derived from CD34^+^ cells and monocytes revealed increased expression of CD45, antigen presentation marker HLA-DR, macrophage marker CD14, and the FcγRI receptor CD64 (a marker for neutrophils and monocytes). Each point in **c**, **e**, **f** represents an individual animal (n = 3 or more per group). Statistical significance was determined using one-way ANOVA with multiple comparisons correction, significance indicating *p < 0.05, **p < 0.01, ***p < 0.001, ****p < 0.0001. In **c-e**, peripheral primary microglial transplant resulted in no brain cell engraftment.

To investigate how ontogeny and environment contribute to human microglial identity, we compared four human myeloid cell populations and two different xenotransplantation approaches combinatorially using a humanized immunodeficient murine model wherein microglia are genetically depleted to allow for full niche availability (*Csf1r*^ΔFIRE/ΔFIRE^ *CSF1*^h/h^ *Rag2*^−/−^ *Il2rg*^−/−^ referred to herein as the hFIRE model)^31^. Employing early postnatal (P2–4) xenotransplantation either directly into the CNS or peripherally (intrahepatic) we transplanted induced pluripotent stem cell derived hematopoietic progenitors (iHPCs), which we have previously shown closely recapitulate primary human microglia when transplanted directly into the CNS^31,32^, or either umbilical cord blood derived CD34^+^ progenitors or CD14^+^ blood monocytes (Fig. 1a, **Extended Data Fig. 1a)**. Of note, the limited cells derived from any single umbilical cord sample required pooling, such that > 100 diverse donor samples were utilized in these studies overall. The field has notably lacked definitive benchmarking for human microglia in xenotransplantation models; we therefore paired these three experimental groups with xenotransplanted primary human microglia^32^ as a novel benchmark for comparison.

We first queried the ability of different myeloid ontogenies and transplantation approaches to attain and fill the CNS. In adult (8–12 week old) mice, brains engrafted with iHPCs, CD34^+^ HSCs, and monocytes resulted in tiled human myeloid cells evenly distributed throughout the brain (Fig. 1b–c). Surprisingly, when primary postnatal microglia were xenotransplanted into the CNS, myeloid cells only engrafted a portion of the brain closest to thalamus, suggesting limited proliferation potential (Fig. 1b–c). With early postnatal peripheral xenotransplantation, we intriguingly identified migrated human myeloid cells in the brain after engraftment with iHPCs, CD34^+^ HSCs, and monocytes, whereas no human myeloid cells were detected after peripheral engraftment of primary microglia (Fig. 1b–c). These findings suggest that different types of myeloid cells exhibit different abilities to fill the CNS myeloid niche.

We next queried the morphology of different myeloid ontogenies, identified based on the human nuclei marker Ku80 and myeloid IBA1 expression (Fig. 1d). Morphological analysis for ramification complexity (skeletonized branches and endpoints), cell size and IBA1 expression, found that primary microglia and iHPCs transplanted in the CNS were larger, more ramified, and expressed higher IBA1 compared to myeloid lineages, CD34^+^ HSCs and monocytes (Fig. 1e). iHPCs that were peripherally transplanted morphologically were more similar to CD34^+^ and monocyte populations, with fewer ramifications than those transplanted in the CNS (Fig. 1e). To further characterize brain myeloid marker expression, we performed marker expression of xenotransplanted human brain myeloid cells by flow cytometry **(Extended Data Fig. 1b)**. Given limited availability and poor proliferation of primary human microglia, insufficient samples were available for flow. Notably, expression of the leukocyte common antigen CD45 was lower in CNS transplanted iHPCs, whereas it was elevated in brain myeloid cells derived from CNS-engrafted CD34^+^ cells and monocytes (Fig. 1f). High CD45 expression in monocytes was consistent with the findings of infiltrating monocytes in murine studies^33^. CD64, a marker for the Fc gamma receptor, was significantly elevated in brain CD34^+^ HSCs and monocytes after peripheral engraftment (Fig. 1f), suggesting that an activated monocytic cells maintain high accessory of inflammatory response regardless of engraftment site^34^. The antigen presentation marker HLA-DR was upregulated in brain myeloid cells derived from CNS-engrafted monocytes, while the monocyte marker CD14 was elevated—by approximately fourfold—in brain myeloid cells derived from CD34^+^ HSCs and monocytes, compared to those derived from iHPCs-engrafted xMGs independent of engraftment site (Fig. 1f). The homeostatic microglia marker CX3CR1 was higher in CNS engrafted iHPCs compared to those from CNS monocyte engraftments **(Extended Data Fig. 1c)**. Collectively, these data suggest that human HSC-derived myeloid cells cannot adopt defining microglial morphological features and display increased expression of monocyte-like, pro-inflammatory and antigen presentation markers compared to yolk sac–derived lineages.

### Distinct gene expression profiles of human myeloid ontogenies in the brain

**Figure 2:**
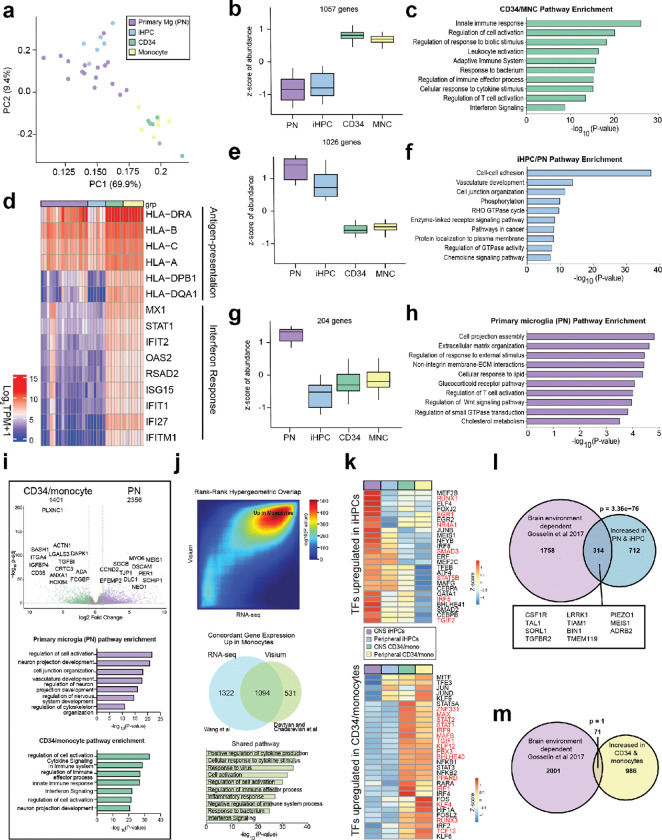
Ontogeny and environment drive transcriptional changes in brain myeloid cells **(a) Principal component analysis (PCA) of RNA-seq from xenotransplanted brain myeloid cells (CD11b**^**+**^**CD45**^**+**^ **cells)** based on 25,537 genes. **(b) Differential gene expression analysis using a likelihood ratio test (LRT) model across four groups.** Overall, 1,057 genes were upregulated in CD34^+^ and monocyte-differentiated brain myeloid cells. Z-score of gene abundance was used to quantify the distance between each data point and the mean, with values expressed in terms of standard deviations. Log2 Fold change > 0.5, padj < 0.05. (c) Pathway enrichment analysis (Metascape) of upregulated genes in CD34^+^ cell- and monocyte-derived brain myeloid cells. (d) Heatmap of highly expressed pathway genes in CD34^+^ cell- and monocyte-derived brain myeloid cells, with genes involved in antigen presentation and interferon response. **(e)** The LRT model identified 1,026 genes upregulated in both PN and iHPCs-derived microglia compared to the CD34^+^ and monocyte-derived brain myeloid cells. The Z-scores of gene abundance was plotted in bar charts to quantify the deviation of each data point from the mean, with values presented as standard deviations. Log2 Fold change > 0.5, padj < 0.05. **(f)** Metascape analysis for pathways enrichment in human postnatal (PN) and iHPCs-derived microglia. **(g)** LRT analysis identifies 204 genes upregulated in PN microglia compared to all other groups. Log2 Fold change > 0.5, padj < 0.05. **(h)** Pathway enrichment analysis (Metascape) of genes upregulated in PN. **(i)** Pairwise comparison between postnatal microglia (PN) and the combined data of CD34^+^ and monocyte-derived brain myeloid cells with Metascape specific pathway enrichment. P-adj < 0.05. Genes with a |log2 fold change| > 1 are considered significantly upregulated or downregulated, representing at least a two-fold increase or decrease in expression. **(j)** Rank-Rank Hypergeometric Overlap (RRHO) analysis revealed strong concordance in the gene signatures of monocyte-derived myeloid cells between our RNA-seq data and the Visium spatial dataset from Davtyan and Chadarevian et al. Based on the RRHO analysis, the most co-upregulated genes based on ranking identified 1,094 overlapping genes visualized by Venn diagram. Pathway enrichment of these shared genes using Metascape highlighted processes related to cytokine production, cell activation, and interferon signaling, with −log10(p-value) > 10. **(k)** Heatmap of transcription factor gene expression across the four groups. Genes marked in red are differentially expressed (|log2FC| > 1, padj < 0.05) between iHPCs- and CD34/monocyte-derived cells in the CNS. **(l)** Venn diagram of the overlap of genes upregulated in postnatal and iHPCs-derived microglia in the CNS (1026, Fig. 2e) overlapped with microglial genes regulated by the brain environment (Gosselin et al^19^). Hypergeometric p value is included. p-adj < 0.05 and Log2FC > 0.5. **(m)** Venn diagram of brain environmentally response genes with those upregulated in CD34 and monocyte derived brain myeloid cells (1057, Fig. 2b), hypergeometric p with p-adj < 0.05 and Log2FC> 0.5. Analysis based on n ≥ 6 samples from all groups except for postnatal microglia (n = 16 derived from Han CZ et al^30^ and Gosselin D et al^19^).

To elucidate transcriptomic changes underlying these distinct ontogenies and environmental conditions, we performed RNA sequencing on isolated myeloid populations (CD11b^+^CD45^+^) 8–12 week after CNS engraftment. We integrated data with RNA sequencing datasets from primary microglia for comparative analysis^31^. Principal component analysis (PCA) revealed that postnatal microglia (PN) clustered closely with iHPCs-derived xMGs in the CNS, while remaining distinct from CD34^+^/monocyte-derived myeloid cells, suggesting a closer transcriptional relationship between PN and iHPCs-derived xMGs after batch correction (Fig. 2a).

To assess significant differences in gene expression across multiple experimental groups, we applied a Likelihood Ratio Test (LRT) model to determine the overall effect of ontogeny on gene expression among brain myeloid cells from the four groups. We identified 1,057 genes that were upregulated (fold change > 0.5, FDR-adjusted *p* < 0.05) in CD34^+^ HSCs and monocyte-derived brain myeloid cells compared to primary PN and iHPCs-derived xMGs in the CNS (Fig. 2b, **Supplementary Table 1)**. These included genes such as *MS4A7*, *MS4A4A*, *GPNMB*, *LGALS3*, *STAT1*, *OAS2*, *HLA-DRA*, and *CD9* **(Extended Data Fig. 2a)**. Gene Ontology (GO) analysis revealed that these genes were significantly enriched in pathways related to immune response, cell activation, and interferon signaling (Fig. 2c). Specifically, genes involved in antigen presentation including *HLA-A, HLA-B, HLA-C, DRA, DQB,* and interferon response genes *MX1, STAT1, IFIT2, and IFITM1,* were significantly upregulated in CD34^+^ and monocyte-derived brain myeloid cells (Fig. 2d). In contrast, 1,026 genes were upregulated (fold change > 0.5, FDR-adjusted *p* < 0.05) in primary PN and iHPCs derived xMGs compared to CD34^+^ cell- and monocyte-derived brain myeloid cells (Fig. 2e, **Supplementary Table 1)**, including homeostatic markers (*TMEM119*), microglial environmentally regulated genes (*BIN1*)^35^, genes implicated in microglial synaptic modulation (*RAB3D*, *DHRS9*, *ADRB2*, *TIAM1*, *ARGLU1*) and the transcription factors *TAL1* and *SMAD*3 **(Extended Data Fig. 2b)**. Analysis of this gene set revealed significant enrichment in processes related to cell-cell adhesion, with prominent terms including vasculature development, cell junction organization, and the chemokine signaling pathway (Fig. 2f). Notably, 204 genes were found to be uniquely upregulated (fold change > 0.5, FDR-adjusted *p* < 0.05) in primary microglia (Fig. 2g, **Supplementary Table 1)**, and these genes were enriched in processes related to cell projection assembly, extracellular matrix organization, and response to external stimuli (Fig. 2h). This expression profile, including genes such as the sodium channel subunit beta-1 (*SCN1B*)^36^ and cadherin-related family member 3 (*CDHR3*)^37^, may reflect the molecular changes underlying the brain connectivity differences between mice and humans.

A comparison of CD34^+^ HSCs and monocytes after CNS engraftment identified only a single significantly differentially expressed gene**, *ECE1***, which was increased in CD34^+^ HSC-derived cells **(Extended Data Fig. 2d)**. ECE1 is essential for the activation of endothelins, peptides that play a critical role in vascular regulation and neural functions^38^. Given this similarity, we combined these groups for a comparative analysis to primary PN and iHPCs derived xMGs, aiming to further elucidate the expression profiles and functional implications in these contexts. Comparison to primary PN microglia found substantial upregulation in pathways related to immune response and cell activation, highlighted by the increased expression of ***LGALS3***and ***DAPK1*** (Fig. 2i). In contrast, genes such as ***MYO6***and ***MEIS1***, which are involved in actin filament-based processes, vasculature development, and CNS development, were significantly downregulated (Fig. 2i). The findings suggest a reduced engagement in neurodevelopmental processes, supporting an inflammatory phenotype in bone marrow derived myeloid cells in the CNS environment^39–42^. Comparison to iHPCs derived xMGs found upregulated genes included ***HLA-DQB1*** and ***TRIM14*(Extended Data Fig. 2c)**, which are associated with antigen presentation and interferon signaling pathways, suggesting a role in immune activation. Conversely, genes related to CNS and brain development, such as ***MEIS1*** and ***KIF27* (Extended Data Fig. 2c)**, were significantly downregulated in CD34^+^/monocytes derived brain myeloid cells, implying a diminished capacity for neuronal support and surveillance in the CNS environment^43,44^. To assess the generalizability of our findings, we compared our bulk RNA-seq datasets with the Visium spatial DEG2 dataset generated by an independent group (Davtvan and Chadarevian et al.), who engrafted adult CD14^+^ monocytes into hFIRE murine brains and performed spatial profiling using Visium. Rank-Rank Hypergeometric Overlap (RRHO) analysis revealed strong concordance between the two datasets, particularly in the upregulated gene signatures of HSC-derived myeloid cells relative to microglia, with consistent enrichment of inflammatory pathways and suppression of homeostatic microglial programs (Fig. 2j). Collectively, these findings demonstrate that the transcriptional programs we identified are robust and conserved across independent studies and further indicate that bone marrow–derived myeloid cells in the brain acquire an immune-focused phenotype distinct from the neuroprotective functions typically attributed to microglia.

To further investigate how ontogeny influences myeloid cell differentiation, we analyzed transcription factor (TF) expression across different myeloid developmental origins engrafted in distinct environments. Several transcription factors—including *RUNX1*, *EGR1*, *SMAD3*, and *NR4A1*—are upregulated in iHPCs-derived cells compared to CD34/monocyte-derived cells following CNS engraftment (Fig. 2k). These transcription factors are known to regulate **TGF-β signaling**^45^ and **NF-κB/p65 activity**^46^, thereby contributing to the maintenance of microglial homeostasis and the suppression of neurotoxic inflammation. However, the upregulated transcription factors (TFs) in CD34/monocyte-derived cells from both engraftment approaches—including ***IRF1***, ***BHLHE40***, ***STAT1***, and ***PPARD*** (Fig. 2k)—are associated with the promotion of **inflammatory responses, lysosomal dysregulation**, and **altered lipid metabolism** in microglia^47–49^.

We previously found that primary human microglia are extraordinarily sensitive to loss of the brain environment, such that expression of more than 2000 genes decreases more than a fourfold within twenty-four hours of culture^19^. Given the differential regulation of transcription factors implicated in the microglial brain environmental response based on myeloid ontogeny, we therefore next queried if genes identified by the LRT model overlapped with this dynamic environmentally responsive gene set. Overlay of the Gosselin brain environment-associated gene set with genes increased in primary PN and iHPCs derived xMGs (from Fig. 2e) revealed a highly significant overlap of **314 homeostatic genes (hypergeometric p = 3.36e-76)**, including *TMEM119* and *CSF1R* (Fig. 2l). In contrast, only **71 genes** (p = 1) overlapped between the genes upregulated in CD34^+^/monocyte-derived cells in the CNS (from Fig. 2b) and the environment-dependent gene set (Fig. 2m). Conversely, we performed RNA-seq on primary monocytes prior to engraftment and overlaid these gene sets to identify if bone marrow derived myeloid cells retained a greater degree of monocytic identity. We found **150 overlapping genes** (p = 0.000652) between the genes upregulated in CD34^+^/monocyte-derived cells and those upregulated in primary **monocytes (Extended Data Fig. 2e)**. In contrast, there were **few overlapping genes** between in vitro monocytes and the genes upregulated in PN microglia and iHPCs-derived xMGs in the CNS **(**p = 0.995, **Extended Data Fig. 2e)**. These results suggest that PN-and iHPCs-derived microglia/myeloid cells are more responsive to brain environmental cues in shaping their transcriptional identity, whereas bone marrow derived myeloid cells retain more monocytic features.

We further examined the impact of environmental context on the same cell type—either iHPCs- or CD34^+^/monocyte-derived cells—engrafted in the CNS versus peripheral engraftment. We observed only modest transcriptional differences within each cell type across environments. **iHPCs-derived cells transplanted peripherally** exhibited enrichment in **DNA damage response pathways** compared to their counterparts in the CNS, while **CD34**^**+**^**/monocyte-derived brain myeloid cells after peripheral engraftment** showed greater association with **collagen activation** and **endothelial cell proliferation pathways** relative to those engrafted in the CNS **(Extended Data Fig. 2f-g)**. Taken together, these findings suggest that the observed differences in gene expression arise from ontogeny-based variations, which are further modulated by brain environmental stimuli.

### Human brain myeloid heterogeneity based on ontogeny and engraftment site

**Figure 3:**
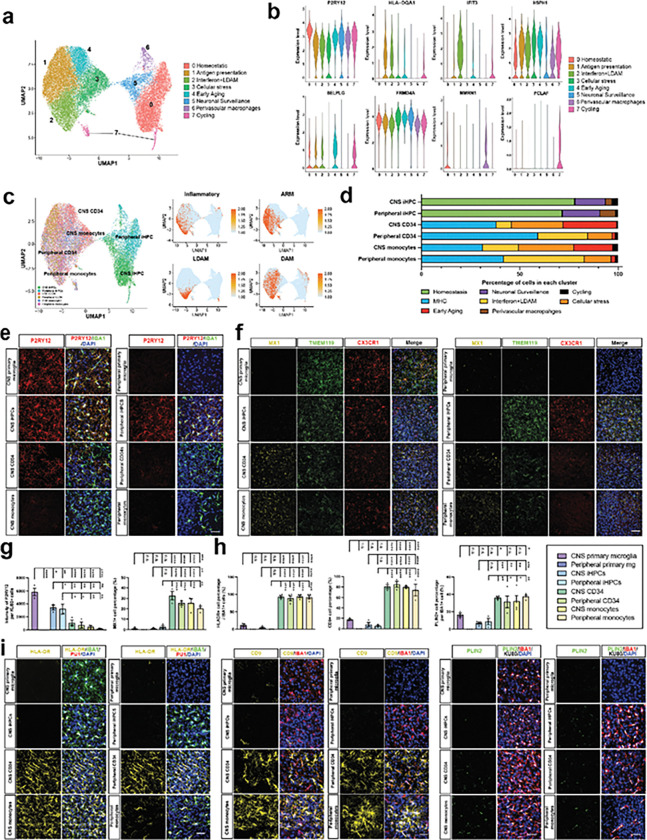
Distinct heterogeneity of xenotransplanted human myeloid cells driven by ontogeny **(a) UMAP plot of xenotransplanted brain myeloid cells.** Visualization of 40,031 CNS myeloid cells derived from adult mice from six xenotransplanted groups (n = 2 biological replicates per group). Clustering was performed using 10 principal components at a resolution of 0.3, with batch effects corrected via Harmony to identify eight total clusters. **(b) Violin plots of cluster-specific gene expression,** representative genes highlight unique features of each cluster. **(c)** The UMAP by sample type divides by ontogeny. Signature genes were scored and aggregated within clusters, revealing enrichment of inflammatory, ARM, LDAM, and DAM signature genes in CD34^+^ cell- and monocyte-derived myeloid cells. **(d) Cluster composition across xenotransplantation groups.** Bar charts depict the percentage of cells in each cluster for each group. **(e)** Immunostaining for the homeostatic microglial marker P2RY12, shows high expression in postnatal primary microglia as well as in iHPCs-engrafted microglia in both CNS and peripheral transplantation. Scale bar = 40 μm. **(f) Immunostaining for homeostatic markers TMEM119 and CX3CR1 as well as interferon marker MX1. Homeostatic markers decreased and interferon response increased in CD34 and monocyte transplantation both in the CNS and peripherally.** Scale bar = 100 μm. (g) Quantification of immunostaining for P2RY12 and MX1. (h) Quantification of immunostaining for HLA-DR, CD9 and PLIN2. **(i)** Immunohistochemistry for HLA-DR, a marker of antigen presentation, PLIN2 as a marker of lipid accumulation, and CD9 as a marker of disease associated macrophages all are increased in CD34 and monocyte transplantations. Scale bar = 40 μm. Bar charts (**g-h**) quantify the percentage of positive transplanted myeloid cells, with each point representing an individual animal (n = 3–4 per group). Statistical significance was evaluated using a one-way ANOVA with multiple comparison corrections. Significance levels are indicated as follows: *p < 0.05, **p < 0.01, ***p < 0.001, ****p < 0.0001.

Microglia represent a highly heterogeneous population composed of distinct subpopulations with diverse functions, differing in their transcriptional profiles, morphological characteristics, and functional roles in CNS development, homeostasis, and response to injury or disease^50^. To understand the gene expression heterogeneity of human myeloid cells in the brain environment, we performed single-cell RNA sequencing (scRNA-seq) on sorted cells from iHPCs-, CD34^+^ HSC- and monocytes-derived brain myeloid cells from adult animals. A total of 40,031 cells were sequenced across all groups, establishing an in-depth analysis of transcriptional profiles at single-cell resolution. After quality filtering, normalization, and Harmony batch correction, clustering analysis identified eight distinct clusters (Fig. 3a). Based on the expression of canonical marker genes, we annotated the clusters as follows: homeostatic microglia (*P2RY12*+), major histocompatibility complex expressing (MHC, *HLA-DQA1*+), interferon and lipid-droplet-accumulating microglia (LDAM) (*IFIT3*+, *LIPA+),* Cellular stress (*HSPH1*+), early aging signature (*SELPLG*+), neuronal surveillance (*FRMD4A*+), perivascular macrophages (*MMRN1*+) and cell cycling (*PCLAF*+) (Fig. 3a–b, **Extended Data Fig. 3a)**. These annotations highlighted distinct functional states within the brain myeloid populations influenced by their ontogeny and environment.

We observed clear separation between **iHPCs- and CD34**^**+**^**/monocyte-derived myeloid cells** after CNS and peripheral transplantation (Fig. 3c). Next, we computed enrichment scores^19,51–57^, finding that bone marrow derived myeloid cells exhibited higher enrichment scores for pathways associated with inflammation, activated response microglia (ARM), LDAM and disease-associated microglia (DAM) (Fig. 3c), indicating an altered cell type toward a pro-inflammatory and disease-responsive state under these conditions. Fractional analysis of cell populations revealed a significant increase in the homeostatic, neuronal surveillance, and perivascular macrophage clusters in iHPCs-derived xMGs compared to CD34^+^ and monocyte-derived brain myeloid cells, suggesting that iHPCs-derived cells may retain more homeostatic and surveillance functions. In contrast, clusters associated with MHC, interferon, LDAM, cellular stress, and early aging were upregulated in CD34^+^ and monocyte-derived brain myeloid cells, indicating a shift toward an immune-responsive phenotype (Fig. 3d). Notably, MHC-associated clusters were upregulated by 0.5-fold, and interferon and LDAM clusters were upregulated by 2-fold in CD34^+^ and monocyte-derived brain myeloid cells after peripheral transplantation compared to those in the CNS. Conversely, cellular stress and early aging signatures were increased by 2-fold and 12-fold, respectively, in CNS-transplanted bone marrow derived myeloid cells compared to their peripherally transplanted counterparts (Fig. 3d). These findings suggest that the initial engraftment into the CNS environment may drive more aging and stress-related features, while initial engraftment into the peripheral environment may promote immune response and antigen presentation. [CG1] [NC2] Our scRNA-seq analysis demonstrated unique transcriptional signatures in brain myeloid cells derived from different ontogenies that is informed by environment.

We also overlaid the Visium datasets from Davtvan and Chadarevian et al. onto our scRNA-seq data. Upregulated microglial genes from their dataset showed greater enrichment in our iHPCs-derived myeloid cells delivered either in the CNS or peripherally **(Extended Data Fig. 3b)**. In stark contrast, genes upregulated in adult monocyte–derived myeloid cells within their Visium dataset were enriched in our peripherally delivered HSC-derived myeloid cells, suggesting that adult monocytes engrafted directly into the CNS exhibit entrainment from the peripheral environment similar to peripherally delivered umbilical cord blood derived cells **(Extended Data Fig. 3b)**. We next validated these findings at the protein level and orthogonally benchmarked to xenotransplanted primary human microglia for additional validation. Expression of the homeostatic marker P2RY12, TMEM119 and CX3CR1 were high in primary microglia after CNS transplantation, and higher in iHPCs regardless of transplantation site than any bone marrow derived myeloid population (Fig. 3e–g). Conversely, expression of the interferon response gene MX1, was markedly elevated in CD34^+^ HSCs and monocyte derived cells regardless of transplantation site (Fig. 3f–g, **Extended Data Fig. 3c)**. Similarly, expression of MHC (HLA-DR), lipid droplets (PLIN2) and the DAM marker CD9 were all markedly elevated in HSC-derived macrophages independent of engraftment site (Fig. 3h–i, **Extended Data Fig. 3c)**. Importantly, **HLA-DR, MX1, CD9**, and **PLIN2** expression were largely absent in **postnatal microglia** and **iHPCs-derived xMGs** within the CNS. This suggests that, in contrast to **CD34**^**+**^**/monocyte-derived xMGs**, iHPCs-derived xMGs maintain a more **homeostatic transcriptional profile** with **minimal signs of immune activation**. Surprisingly, peripheral transplantation of iHPCs yielded similar expression of P2RY12, CX3CR1 and TMEM119 comparable to that seen with CNS engraftment, despite peripheral entrainment, distinctly different from peripheral engraftment of bone marrow derived myeloid cells. Noted within the scRNA-seq was an increase in perivascular macrophages from peripheral iHPCs transplantation (Fig. 3d). Quantitation of LYVE^+^ within cortical perivascular areas found that peripheral myeloid cells have reduced capacity to differentiate to perivascular macrophages, with a trend towards higher perivascular macrophages from peripheral iHPCs transplantation **(Extended Data Fig. 3d)**. Collectively, these results indicate that **brain myeloid cells derived from different developmental origins** exhibit **distinct protein expression profiles**, which shape their **immune activation potential and homeostatic state in an otherwise healthy brain environment**.

### Human brain myeloid cells exhibit unique transcriptional differences from murine models

**Figure 4:**
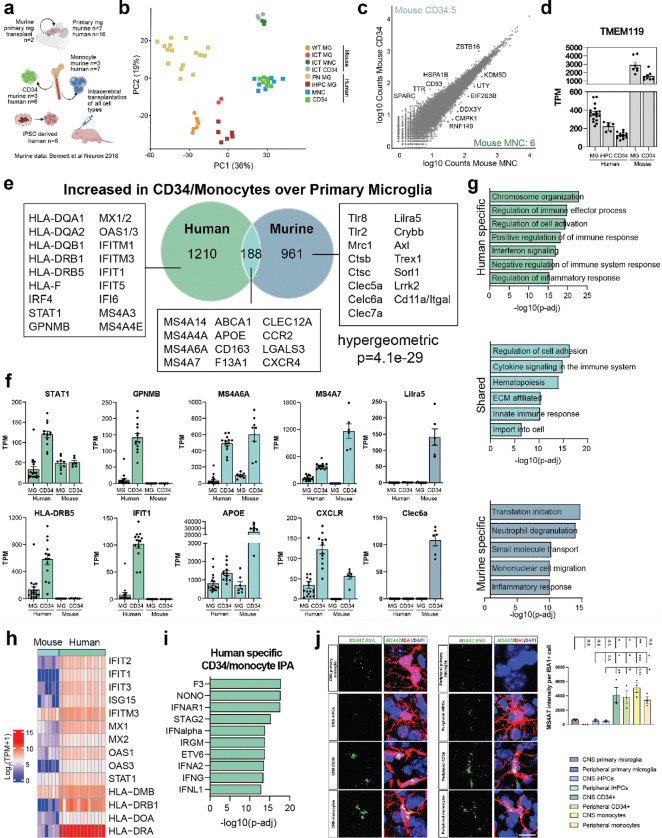
Distinct and shared human and mouse brain myeloid cell signatures **(a)** Schematic overview comparing human primary and xenotransplanted samples to a murine dataset^26^. **(b)** Principal Component Analysis (PCA) of human and mouse samples based on gene expression profiles. Mouse samples include WT MG (wild-type microglia), ICT MG (intracerebral transplanted microglia), ICT MNC (intracerebral transplanted monocytes), and ICT CD34 (intracerebral transplanted bone marrow). **(c)** Scatter plot visualizing differential gene expression in mouse CD34^+^ and monocytes-derived brain myeloid cells represented by log10 counts. Five genes—*ZBTB16*, *HSPA1B*, *CD93*, *TTR*, and *SPARC*—were significantly upregulated in mouse CD34^+^ cells. In contrast, six genes—*KDM5D*, *UTY*, *EIF2S3B*, *DDX3Y*, *CMPK1*, and *RNF149*—were upregulated in mouse monocytes-derived brain myeloid cells. **(d)** Transcript per million (TPM) values of *TMEM119* are shown across human microglia (MG), xenografted microglia (xMG), CD34^+^ cells, and mouse MG and CD34^+^ cells. Each group includes at least six biological replicates (*n* ≥ 6). **(e)** Venn diagram overlap of genes upregulated in CD34^+^ and monocyte-derived brain myeloid cells relative to primary microglia across human and mouse samples. It reveals distinct and shared gene expression patterns. Log2Foldchange > 1 and p-adj < 0.05. **(f)** Representative species specific and shared genes with high expression in human or mouse CD34^+^ and monocyte-derived brain myeloid cells, as well as the overlapping genes shared between human and mouse. **(g)** Metascape enrichment analysis performed on the genes upregulated in CD34^+^ and monocyte-derived brain myeloid relative to primary microglia that are human specific, murine specific, and shared overlapping genes. **(h)** Heatmap of key differentially expressed genes between human and mouse CD34^+^ and monocyte-derived brain myeloid cells. **Human orthologs displayed, murine orthologs: *Ifit2, Ifit1, Ifit3, Isg15, Ifitm3, Mx1, Mx2, Oas1a, Oas3, Stat1, H2-Dmb1, H2-Ab1, H2-Oa, H2-Aa*.** **(i)** Ingenuity Pathway Analysis (IPA) of the upstream regulators of human-specific CD34^+^ and monocyte-derived brain myeloid cells. **(j) RNAscope for *MS4A7***in IBA1-positive human brain myeloid cells with quantification of *MS4A7* intensity. Scale bar = 15 μm. Statistical significance was determined using one-way ANOVA with multiple comparison corrections. Significance levels are indicated as follows: *p < 0.05, **p < 0.01, ***p < 0.001, ****p < 0.0001.

Numerous studies have examined the fate of murine bone marrow derived macrophages in the brain environment, but none have queried the species specificity between murine and human models. Murine studies have concluded that peripheral myeloid cells cannot take on a full yolk sac microglial phenotype, exhibiting reduced expression of homeostatic markers and microglial transcription factors^23,27^. To assess the impact of species differences on myeloid cell characteristics, we integrated a comparable murine dataset from Bennett et al^27^ with our human data, asking which features of the peripheral myeloid response to the brain environment is shared and which is species-specific. This murine dataset included primary murine microglia (n=7), transplanted murine microglia (n=2), bone marrow CD34^+^ (n=3), and blood monocytes (n=3) all introduced via intracerebral transplantation (ICT) which was compared to the human specific xenotransplant datasets generated in this study (Fig. 4a). A PCA plot of orthologs identified six distinct groups, highlighting the PC1 separation between microglia and peripheral bone marrow derived cells across both human and mouse samples (Fig. 4b). Similar to human, only a few differentially expressed genes were identified between mouse CD34^+^ cells (5 genes) and monocytes (6 genes) transplanted into the brain environment (Fig. 4c). Homeostatic marker genes such as *TMEM119* (Fig. 4d) were higher in primary microglia than peripheral bone marrow derived cells, although levels were species-specific.

We next compared the differentially expressed genes by species, identifying genes that were increased in CD34^+^ and monocytes in the brain when compared to microglia with similar cutoffs (FC > 2, padj < 0.05) and asking whether there is species specificity in the brain myeloid signature for bone marrow derived macrophages. We observed substantial differences, finding that 1,210 genes were uniquely identified in the human dataset, including *HLA*, *STAT1*, *MX1/2*, and *IFITM1/3*, while 961 genes were specific to the murine data, including *Mrc1*, *Clec7a*, and *Axl* (Fig. 4e). Overlap was statistically significant (hypergeometric p = 4.1e-29) with 188 common genes, including *MS4A7*, *APOE*, and *ABCA1* being increased in both species (Fig. 4e, **Supplementary Table 2)**. These data indeed show that interferon genes (*STAT1*, *IFIT1*), antigen presentation (*HLA-DRB5*) were increased only in human CD34 and monocytes in the brain. Some genes that had been postulated as potential markers of peripheral monocytes (*Lilra5*, *Clec6a*, *Clec7a* and *Tlr8*) were murine specific (Fig. 4f, **Extended Data Fig. 4a-b)**.

Murine specific genes increased in CD34^+^ and monocytes-derived brain myeloid cells were significantly enriched in pathways related to translation initiation, degranulation and inflammation response (Fig. 4g). Ingenuity Pathway Analysis (IPA) to identify enriched upstream regulators and canonical pathways enriched in murine CD34^+^ and monocytes identified homeostatic regulators such as ERG and ERBB2, and pathways related to hematopoiesis and metabolism **(Extended Data Fig. 4c-d)**.

Pathway analysis of human-specific genes increased in bone marrow derived myeloid cells found enrichment in pathways associated with inflammation and cell activation, and interferon signaling (Fig. 4g). Indeed, the differences between murine and human bone marrow myeloid cells in interferon response and antigen presentation gene expression were marked (Fig. 4h). IPA identified numerous interferon associated genes such as IFNAR1, IFNalpha, and IRGM, and pathways associated with cytokine production and activation (Fig. 4i, **Extended Data Fig. 4e)**. [CG3] [WJ4] To orthogonally validate these substantial species-specific differences in myeloid transcription, we integrated data from a second study that identified a core of gene set identifying murine peripheral myeloid identity using multiple bone marrow transplant approaches from Cronk et al.^23^. This comparison also noted minimal species specificity, with little conservation of the gene signature in our human xenotransplanted samples **(Extended Data Fig. 4f)**.

Shared markers of bone marrow ontogeny between species included *MS4A* family members, and common upregulation of *ApoE* in bone marrow derived myeloid cells (Fig. 4e–f). Indeed, RNAscope for *MS4A7* and *APOE* confirmed this finding in human myeloid cells, revealing high expression in CD34^+^ and monocytes-derived brain myeloid cells after both CNS and peripheral transplantation, compared to primary transplanted microglia and iHPCs-derived cells (Fig. 4j, **Extended Data Fig. 4g)**. Collectively, these findings highlight fundamental divergences in the transcriptional programs of CNS-engrafted myeloid cells across species, with conservation of some markers of peripheral myeloid ontogeny such as MS4A family members, but substantial differences with a human specific antigen presentation and interferon activation not identified in multiple comparable murine studies.

### Transcription factors networks driving brain myeloid cell fates

**Figure 5:**
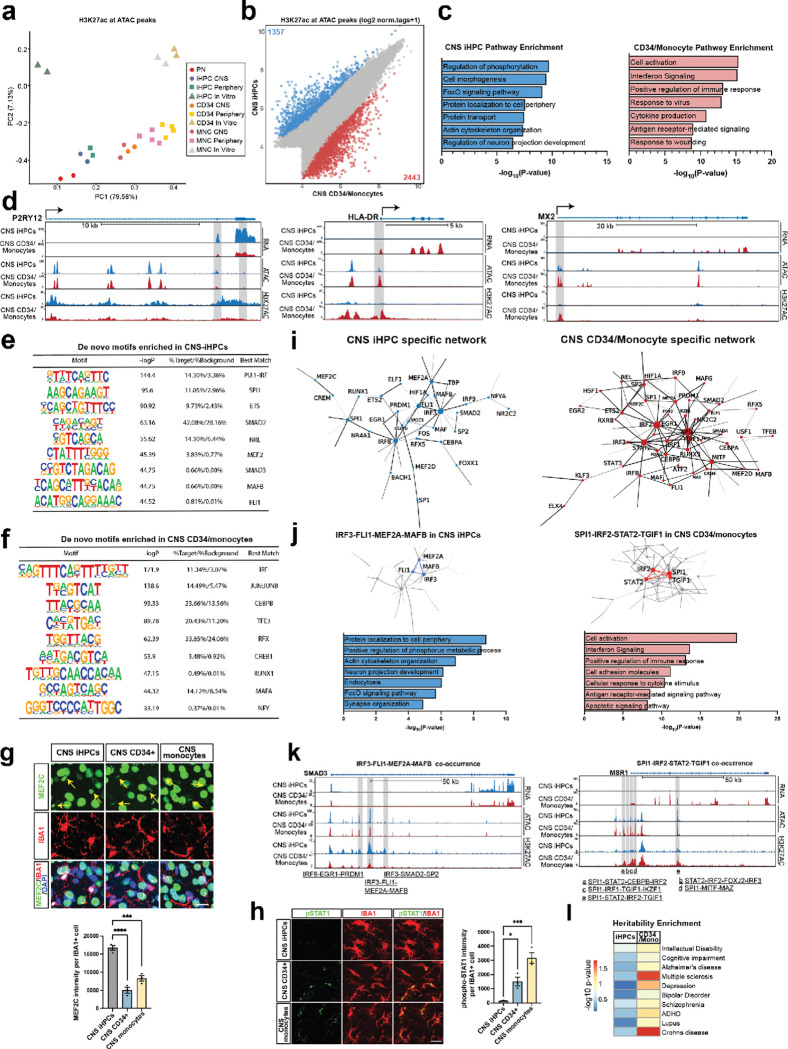
Epigenetic landscape and transcriptional network regulation of myeloid cells in the brain environment **(a)** Principal Component Analysis (PCA) plot of ATAC-seq data annotated with H3K27ac ChIP-seq signals, illustrating the active enhancer landscape across human primary microglia, myeloid cells derived from iHPCs, CD34^+^ cells, and monocytes from both the CNS and periphery, as well as in vitro cultured iHPCs, CD34^+^ cells, and monocytes. **(b)** ATAC-seq analysis annotated for active H3K27ac, combining CD34 and monocytes into a single analysis group to compare to iHPCs (n = 2–4 biological replicates per group). Differentially acetylated regions (FC > 2, padj < 0.05) enriched in CNS iHPCs (blue) and CNS CD34 and monocytes (red) are colored. **(c)** Metascape pathway enrichment analysis of the nearest genes associated with differential peaks upregulated in iHPCs-derived (1,357 peaks, blue) and CD34/monocyte-derived (2,433 peaks, red) for promoter/promixal regulatory regions (ATAC-seq peaks <1,000 bp from the transcription start site [TSS]). Bar plots display pathways with a −log_10_(p-value) greater than 5**.** **(d)** UCSC genome browser tracts showing expression and corresponding ATAC-seq and H3K27ac ChIP-seq data from iHPCs (blue) and CD34^+^ cells/monocytes (red) engrafted in the CNS environment. *P2RY12* marks genes upregulated in iHPCs-derived xMGs, while *HLA-DR* and *MX2* exhibit higher peaks associated with upregulated genes in CD34/monocyte-derived brain myeloid cells, with differential peaks highlighted in grey. **(e)** De novo motif enrichment analysis for differentially acetylated ATAC peaks in iHPCs-derived xMGs using genomic background. **(f)** De novo motif enrichment analysis for differentially acetylated ATAC peaks in CD34 and monocyte-derived CNS myeloid cells using genomic background. **(g)** Immunofluorescence for the transcription factor MEF2C. Yellow arrows indicate MEF2C-positive nuclei co-localized with IBA1^+^ cells with quantification. Quantification of MEF2C (n = 3 biological replicates per group compared by one-way ANOVA with multiple comparison correction). Scale bar = 15 μm. **(h)** Phosphorylated-STAT1 expression in transplanted myeloid cells. The intensity of phosphorylated-STAT1 is compared across groups. Quantification of pSTAT1 (n = 3 biological replicates per group by one-way ANOVA with multiple comparison correction). Scale bar = 15 μm. **(i)** TIMON analysis of differentially acetylated ATAC peaks. Nodes represent transcription factor (TF) motifs, while edges indicate significant co-occurrence (p < 0.001) between TF pairs. Blue nodes correspond to TF networks enriched in iHPCs-derived xMGs, and red nodes represent those enriched in CD34/monocyte-derived brain myeloid cells. The size of the nodes reflects their connectivity (degree), with nodes having a degree greater than 10 labeled. Co-occurring motifs with nodes (n ≥ 2) are also labeled. **(j)** The cliques identified in the iHPCs-derived (blue, top) and CD34/monocytes-derived brain myeloid cells network (red, top) highlight key transcription factor (TF) interactions. The representative cliques, IRF3-FLI1-MEF2A-MAFB (blue), were predominantly observed in the iHPCs-derived xMGs, with frequent co-occurrence of TF nodes in the network connectivity. SPI1-IRF2-STAT2-TGIF1 cliques (red) were detected in the upregulated peaks of the CD34/monocytes-derived brain myeloid cell network. The bar chart at the bottom shows the Gene Ontology (GO) terms associated with enhancers of the IRF3-FLI1-MEF2A-MAFB (blue) and SPI1-IRF2-STAT2-TGIF1 (red) cliques, with −log_10_(p-value) greater than 4. **(k)** UCSC browser tracks show that *SMAD3* is regulated by the co-occurring motifs IRF3-FLI1-MEF2A-MAFB. In contrast, *MSR1* is upregulated by the SPI1-IRF2-STAT2-TGIF1 motifs. **(l)** Linkage disequilibrium score regression analysis showing the enrichment of disease-associated risk alleles in xMGs derived from iHPCs and CD34^+^/monocyte precursors, based on differential H3K27ac ChIP-seq peaks. Enrichment significance is represented as −log_10_(p-value), indicated by the color scale.

To investigate the transcriptional mechanisms underlying these human specific brain myeloid phenotypes, we next characterized the epigenetic landscape, performing assays for transposase-accessible chromatin (ATAC-seq), alongside chromatin immunoprecipitation (ChIP-seq) for histone H3 lysine 27-acetylation (H3K27ac) a marker of active promoters and enhancers. A PCA of differentially acetylated transposase accessible chromatin regions between samples revealed four distinct groups: iHPCs-derived xMGs engrafted in the CNS and peripherally clustered with primary microglia, CD34^+^ HSCs and monocyte-derived brain myeloid cells with both transplantation approaches, iHPCs cultured in vitro, and in vitro CD34^+^ HSCs and monocytes (Fig. 5a). The separation observed along PC1 suggests that the primary source of variation in chromatin accessibility is attributed to the effect of ontogeny, differentiating yolk sac from peripheral myeloid cells. In contrast, the separation along PC2 appears to be driven by environment, distinguishing ex vivo from in vitro samples. These findings highlight substantial changes in chromatin accessibility and enhancer activity, influenced by both environmental cues and developmental origin. Given the transcriptional similarity between CD34^+^ HSCs-derived and monocyte-derived brain myeloid cells, we grouped them together for analysis. We identified 1,357 (blue) differential peaks upregulated in iHPCs-derived xMGs and 2,443 peaks (red) upregulated in CD34^+^/monocyte-derived brain myeloid cells (Fig. 5b). GO analysis revealed that upregulated peaks in iHPCs derived xMGs were enriched in the regulation of phosphorylation, cell morphogenesis and regulation of neuron projection development (Fig. 5c). Conversely, the peaks enriched in bone marrow derived myeloid cells were near genes implicated in interferon signaling, cytokine production and antigen receptor-mediated signaling pathways (Fig. 5c). This included loss of acetylation at enhancers and promoters at homeostatic genes (*P2RY12*) and gain at antigen presenting (*HLA-DR*) and interferon (*MX2*) genes in bone marrow derived myeloid cells (Fig. 5d) (FC>2, FDR<0.05). These findings were mirrored by peripherally transplanted myeloid cells isolated from the brain environment **(Extended Data Fig. 5a)**, with differentially regulated chromatin in bone marrow derived myeloid cells occurring near genes implicated in interferon signaling, cytokine signaling, and the positive regulation of immune responses **(Extended Data Fig. 5b-c)**. These findings suggest that ontogeny specifies the response to the brain environment in a gene-specific manner.

We next sought to identify the lineage determining and signal dependent transcription factors associated with active enhancers across groups, performing *de novo* motif analysis on H3K27ac-marked ATAC peaks. When comparing groups, we found significant enrichment in iHPCs derived xMGs for PU.1, SMAD, MAFB, and MEF2 motifs (Fig. 5e). When compared to peripherally transplanted iHPCs, we identified shared motifs—such as NRL, MAFB, and ETS **(Extended Data Fig. 5d)**. ETS family factors play a pivotal role in regulating myeloid cell development, differentiation, and immune function by controlling the expression of key cytokines, chemokines, and surface receptors such as CSF1R in macrophages^58,59^. SMAD is a key signal determining transcription factor involved in TGF-β signaling, which promotes a quiescent microglial phenotype within the CNS^60^. Lastly, MEF2C has been shown to suppress microglial overactivation and prevent neuronal loss^61^, supporting the maintenance of microglial homeostasis. This process is essential for ensuring effective immune surveillance and response at appropriate times and specific brain regions^62,63^. On the other hand, in CD34^+^ HSCs- and monocyte-derived brain myeloid cells, we observed enrichment of transcription factors such as *IRF*, *AP-1*, and *MITF* family members (Fig. 5f), which was mirrored by bone marrow derived myeloid cells after peripheral transplantation **(Extended Data Fig. 5e)**. These transcription factors have been implicated in promoting hyperinflammation and exaggerated phagocytic activity and are associated with the neurotoxicity^64–66^.

We integrated data from the human postnatal microglia dataset^31^ and compared cell types across the CNS environment, observing loss of MEF2 motifs and enrichment of IRF1, TFE3, RUNX, MAF, and AP-1 showed enrichment in bone marrow derived myeloid cells in the brain environment **(Extended Data Fig. 5f)**. This pattern suggests a loss of homeostasis alongside an increase in inflammatory responses, cellular stress, and lysosomal activity. Moreover, we analyzed the same cell types in different environments: CNS or peripheral engraftment and compared to in vitro (dish) to investigate the ontological effects on microglial differentiation. **Motif analysis** revealed that iHPCs are enriched for BACH2 and GATA6 motifs, becoming more driven by MEF2 and SMAD in the brain environment, whereas CD34 and monocytes were driven by AP-1 and became MAF and IRF driven in the brain environment **(Extended Data Fig. 5f)**. We validated the loss of MEF2 and enrichment of IRF signaling, identifying reduced MEF2C protein expression (Fig. 5g) and elevated phosphorylated STAT1 (Fig. 5h) after CNS transplantation of CD34^+^ HSCs and monocytes compared to iHPCs derived xMGs. These findings were mirrored after peripheral transplantation of these three cell types **(Extended Data Fig. 5g-h)**. Together, these findings suggest that the interplay between cellular ontogeny and environmental cues drives the establishment of distinct epigenetic landscapes, ultimately shaping cooperative transcription factor networks.

We next applied a published validated computational framework for identifying nonoverlapping high-confidence motifs from active enhancers to build a co-occurrence network, TIMON (Transcription factor Interaction Module Network)^31^. Using this analysis, we uncovered lineage and signal dependent transcription factors and their significant interactions that govern the epigenetic regulation of brain myeloid cells derived from different developmental origins. The iHPCs-associated network was enriched for transcription factors involved in maintaining homeostatic microglial identity and quiescence, such as *MEF2*, *IRF* and *SMAD*, whereas the CD34^+^/monocyte-derived network showed a predominance of TFs linked to inflammatory activation and immune responsiveness, including *STAT*, *IRF*, and *MITF* (Fig. 5i). We sought to identify specific cliques (> 2 interconnected nodes) governing the fate of different myeloid ontogenies in the brain environment. Within iHPCs derived microglia, the IRF3–FLI1–MEF2A–MAFB clique was prominent. *SMAD3* is a target gene regulated by the IRF3–FLI1–MEF2A–MAFB clique, with intronic enhancers containing combinations of these motifs as well as SMAD2 motifs (Fig. 5k).

This contrasted with bone marrow derived myeloid cells where the SPI1–IRF2–STAT2–TGIF1 clique was dominant (Fig. 5j). *MSR1* was identified as a target gene of the SPI1–IRF2–STAT2–TGIF1 clique, with five distinct genomic regions showing diverse motif compositions associated with its regulation (Fig. 5k). *MSR1* encodes a macrophage scavenger receptor binding to oxidized-LDL, Aβ and APOE^67–69^—processes that are closely linked to neurodegeneration. IRF3 suppresses inflammatory signaling pathways in macrophages to prevent viral pathogenesis^70^, whereas IRF1/2 amplifies inflammatory signals by regulating nitric oxide synthase induction in macrophages^71^, highlighting the distinct functional characteristics of inflammatory factors on xenotransplant cells. Genes regulated by the IRF3–FLI1–MEF2A–MAFB axis were enriched in pathways related to homeostasis and neuronal development (Fig. 5j). In contrast, GO analysis revealed that the SPI1–IRF2–STAT2–TGIF1 axis, associated with upregulated peaks in bone marrow derived myeloid cells, regulates genes involved in inflammation, interferon signaling, and antigen receptor-mediated signaling (Fig. 5j). We furthermore assessed disease-associated risk allele enrichment using differential H3K27ac ChIP-seq peaks from the three brain myeloid populations via linkage disequilibrium score regression analysis. Peaks from CD34^+^/monocyte-derived brain myeloid cells showed preferential enrichment for risk loci associated with multiple sclerosis, Alzheimer’s disease, intellectual disability, and depression in comparison to iHPCs peaks (Fig. 5l). These findings identify key transcription factor networks active in HSCs-derived myeloid cells pertinent to neuroinflammation and neurodegeneration.

### Human bone marrow derived myeloid cells trigger astrogliosis, demyelination and synaptic loss

**Figure 6:**
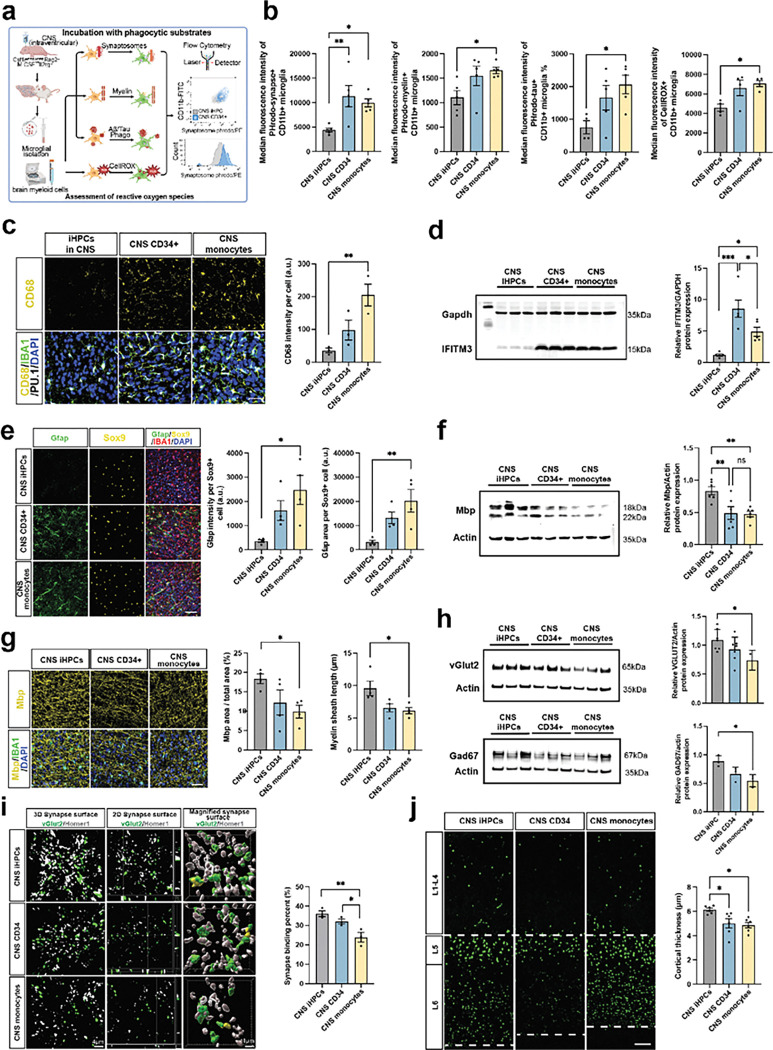
CNS impact of human myeloid cell ontogeny **(a)** Schematic illustration of brain myeloid phagocytic activity and reactive oxygen species. **(b)** Brain myeloid cells were incubated with fluorescently labeled synaptosomes, myelin, Aβ, tau, particles or CellROX, followed by staining for CD11b. Positive cells were identified by flow cytometry, and median fluorescence intensity (MFI) was quantified. **(c)** CD68 intensity was measured within IBA1^+^ cells, revealing higher expression of CD68 in monocyte-derived myeloid cells. Each dot represents an individual animal (n = 3). Scale bars = 40 μm. **(d)** Western blot for IFITM3 expression from human myeloid cell engrafted brains lysates. IFITM3 expression (15 kDa) was normalized to Gapdh (35kDa), and relative protein levels showed an increase in CD34^+^ cell- and monocyte-engrafted brains. **(e)** Immunofluorescence for Gfap in the cortex region of xenotransplanted brains. Gfap intensity and area were quantified and normalized to the number of Sox9-positive cells. Scale bar = 40 μm. **(f)** Western blot of brain lysates was performed to assess myelin basic protein (Mbp) expression in xenotransplanted mice. Mbp levels (15–20 kDa) were normalized to Actin (35 kDa). **(g)** Immunofluorescence for the myelin marker myelin basic protein (Mbp) in cortical areas from xenotransplanted mice. Mbp-positive area relative to the total image area and myelin sheath length were quantified. Scale bar = 40 μm. **(h)** Western blot analysis of excitatory (vGlut2) and inhibitory (Gad67) neuronal markers associated with synaptic function and plasticity, normalized to actin. **(i)** Synaptic connectivity was evaluated by quantifying the interaction between the presynaptic marker vGlut2 (green) and the postsynaptic marker Homer1 (grey). Both 3D and 2D synaptic structures were visualized and the percentage of bound synapses (indicated by yellow on the magnified synaptic surfaces) was calculated by normalizing to the total number of postsynaptic sites (grey). **(j)** Cortical thickness was assessed using the layer 5 / 6 marker Ctip2 in xenotransplanted mice. Cortical thickness was measured in micrometers (μm). Scale bar = 200 μm. Each point represents an individual animal (n = 3–6 per group). Statistical significance assessed by one-way ANOVA with multiple comparison corrections. Significance is indicated as follows: *p < 0.05, **p < 0.01, ***p< 0.001.

Microglia at homeostasis impact diverse cell types through synaptic pruning, myelin clearance, and growth factor secretion such that microglial dysfunction is a critical contributor in neurodegenerative disease. How human bone marrow derived myeloid cells influence brain health, whether protective or detrimental, is unknown and a matter of debate^72–74^. To begin to address this question, we queried the phagocytic potential of human brain myeloid cells after 8–12 weeks of residence in the murine brain to diverse phagocytic stimuli including synaptosomes, myelin, and neurodegenerative specific stimuli coupled with measurements of reactive oxygen species (Fig. 6a). We found that human monocytes, and to a lesser degree human CD34^+^ HSCs, resident in the murine brain exhibited increased phagocytosis of synaptosomes, myelin, and tau (Fig. 6b). Phagocytosis of Aβ1–42 did not reach statistical significance **(Extended Data Fig. 6a)**. Interestingly, measurement of cellular reactive oxygen species (CellROX) was also elevated in bone marrow derived myeloid cells engrafted in the CNS (Fig. 6b).

These results suggest that bone marrow derived myeloid cells are hyperphagocytic, a feature of disease-associated microglia^75,76^, which can lead to detrimental effects, disrupting neural circuit balance, contribute to neuroinflammation, and potentially accelerate neurodegeneration. CD68 is a transmembrane glycoprotein that marks lysosomes and endosomes in macrophages, with higher levels being associated with increased phagocytic activity^77^. We observed that brain engrafted monocytes exhibited significantly higher CD68 intensity compared to those derived from iHPCs, and that CD34^+^ cells exhibited an intermediate phenotype (Fig. 6c, **Extended Data Fig. 6b)**. Peripherally engrafted monocytes and CD34^+^ HSCs exhibited a similar pattern but notably at nearly 10-fold higher levels than for CNS engraftment of the identical cell types, suggesting that the periphery environment may particularly influence lysosomal characteristics in myeloid cells **(Extended Data Fig. 6c-d)**. We also found elevated levels of PICALM, a key regulator of clathrin-mediated endocytosis, in CD34^+^ and monocyte-derived brain myeloid cells, suggesting elevation in the engulfment and uptake of extracellular materials **(Extended Data Fig. 6e)**. Coupled with and building on this hyperphagocytic phenotype in bone marrow derived brain myeloid cells is the transcriptomic data suggestive of interferon pathway cytokine release. Activation of phagocytic brain myeloid cells induces the release of various cytokines and chemokines, which regulate the immune response by recruiting additional immune cells to sites of infection or inflammation, thereby intensifying the immune response^78,79^. We performed a multiplex 80 cytokine array in brain lysates from human myeloid engrafted brains, finding that osteopontin (SPP1) levels were significantly elevated in monocyte-derived brain myeloid cells **(Extended Data Fig. 6f)**, but the assay lacked sensitivity to identify other differential cytokines. Indeed, immunostaining identified increased microglial staining for SPP1 in bone marrow derived human myeloid cells after transplantation **(Extended Data Fig. 6g)**. Elevations in SPP1 have been identified in numerous neurodegenerative disease contexts in acute and chronic neuroinflammation, as a marker of CNS injury^80,81^. To identify interferon pathway activation, we measured whole brain IFITM3 (Interferon-Inducible Transmembrane Protein 3), an interferon-stimulated protein that can be expressed diverse brain cell types. Increased levels of IFITM3 were detected in brain from CD34^+^ HSCs- and monocyte-engrafted brains, suggesting a potential for neurodegeneration^82^ in bone marrow derived myeloid transplanted brains (Fig. 6d).

Given these data, we hypothesized that xenotransplanted bone marrow derived myeloid cells from CD34^+^ and monocytes ontogenies are impaired in their ability to effectively contribute to the maintenance and regulation of other brain cell types, such as astrocytes, oligodendrocytes, and neurons. To address this, we first quantitated cortical astrogliosis, using Gfap relative to total Sox9^+^ astrocytes. This showed significant increases in astrogliosis in monocyte transplanted animals, with CD34^+^ cells exhibiting an intermediate phenotype (Fig. 6e, **Extended Data Fig. 6h)**. Second, we observed an overall reduction in myelin basic protein (Mbp), suggesting impaired myelination or excessive myelin removal in brains engrafted with CD34^+^- or monocyte-derived human myeloid cells (Fig. 6f). To orthogonally validate, we quantitated cortical Mbp density—calculated as the ratio of Mbp-positive area to total area—which was significantly reduced in monocyte transplanted brains compared to those engrafted with iHPCs-derived xMGs (Fig. 6g). This signifies that CD34^+^ and monocyte-derived brain myeloid cells may impair the differentiation and survival of oligodendrocytes, potentially disrupting the formation of myelin sheaths^4^, or may excessively remove myeloid leading to oligodendrocyte damage.

In addition to their interactions with other glia, microglia play a crucial role in neuronal pruning and in response to neural activity, ensuring proper synaptic connectivity by eliminating excess or inactive synapses^83^. To assess synapse numbers, we quantitated expression of the excitatory synaptic marker vGlut2 and the inhibitory synaptic marker Gad67, a key organizer of GABAergic synapses. In human monocyte–engrafted brains, both synaptic were significantly decreased, indicating a disrupted excitatory and inhibitory synaptic signaling (Fig. 6h). We also observed a reduced colocalization of the presynaptic marker vGlut2 with the postsynaptic marker Homer1, suggesting potential disruption of synaptic communication (Fig. 6i). Disruption of key processes such as astrogliosis, myelination, and synaptic pruning can significantly impact cortical structure and integrity. Using Ctip2, a marker for cortical layer 5 and 6 neurons, we observed a reduction in total cortical thickness in CD34^+^ and monocyte engrafted brains compared to those engrafted with iHPCs-derived xMGs (Fig. 6j). Notably, layers 5 and 6 are essential for integrating inputs from deep cortical regions and relaying information to other brain areas and exhibited marked thinning **(Extended Data Fig. 6i)**. These findings suggest that, unlike iHPCs-derived xMGs, brain myeloid cells derived from CD34^+^ HSCs and monocytes are either less effective in maintaining structural integrity and supporting cellular communication essential for brain health or actively cause neuronal cell death. Instead, bone marrow derived macrophages exhibit a hyperphagocytic phenotype and heightened inflammatory activity in the brain environment. This further underscores the critical role of myeloid cell ontogeny in shaping the microenvironment, as these cells are important for preserving proper cortical architecture and intercellular interactions.

## Discussion

Here we describe the brain myeloid differentiation potential of diverse human yolk sac and hematopoietic stem cells, and the influence of CNS as opposed to peripheral delivery on that differentiation fate. Previous studies have queried murine myeloid differentiation potential, identifying that murine peripheral myeloid cells in the brain environment fail to fully acquire homeostatic microglial transcriptional states. However, murine peripheral myeloid cells in these studies have not been associated with overtly injurious cell states or deleterious effects on the brain, leading to the hypothesis that replacement of pathogenic embryonically derived microglia is an attractive therapeutic goal^6,27–29,84^ in diverse neurodegenerative diseases.

We applied a humanized murine xenotransplantation model to delineate the role of ontogeny in human brain myeloid differentiation. While HSCs-derived CD34 progenitor cells and monocytes acquired many features of resident microglia, we unexpectedly observed a dramatic inflammatory response that was restricted to bone marrow derived myeloid progeny. Notably, peripheral myeloid cells exhibited a distinct transcriptional pattern characterized by lower levels of microglial homeostatic markers (P2RY12, TMEM119, CX3CR1) and higher expression of genes related to antigen presentation (MHC), interferon-associated signatures (IFITM3, MX1, STAT1), and lipid metabolism (APOE, NR1H3), indicative of a more disease-associated phenotype. These transcriptional changes were consistent with the Visium spatial findings of Davtvan and Chadarevian et al. Consequently, this inflamed myeloid state was paired, at young adult murine ages, with astrogliosis, demyelination, and synaptic loss altogether culminating in cortical thinning.

An essential area that has been lacking in human xenotransplantation models is benchmarking. Despite multiple publications describing engraftment of iPSC derived hematopoietic progenitors, showing these reasonably approximate primary human microglia with features reminiscent of yolk sac ontogeny^31,32,52,85^, transplantation of primary human microglia has been lacking. We unexpectedly observed that primary human microglia lack the ability to fill the entire brain niche during a time when other myeloid cell types including iPSC microglia readily filled the niche. Notably primary microglia did exhibit robust homeostatic marker expression and morphology. These data further support the yolk sac reminiscent ontogeny of iPSC derived hematopoietic cells after CNS transplantation and starkly contrast with the differentiation potential and inflammatory phenotype of bone marrow derived myeloid cells.

We compared our human datasets with multiple published murine brain monocyte studies and gene signatures to identify the species specific and shared transcriptional signatures. Key shared signatures in both mouse and human brain monocytes include *Ms4a7*, *ApoE*, and *Ms4a4a*. However, notably the antigen presentation and interferon activation were human specific. These differences are primarily species-dependent, as approximately 270 genes in human monocytes and 550 genes in mouse monocytes were differentially expressed by 2-fold or more, with over 130 differences conserved across species, resulting in the opposite expression of certain surface markers, such as CD36, CD9, and TREM-1^86^. Notably, mouse monocytes exhibit a prominent PPARγ signature absent in humans, and both species show contrasting receptor patterns involved in phagocytosis^86^, which may contribute to distinct species-specific monocyte behaviors after CNS transplantation. One limitation to these models is that the brain environment throughout is murine, such that some growth factors and signaling molecules will not signal appropriately between species. These findings may reflect evolutionary adaptations to distinct CNS environments or differences in myeloid tolerance between species, underscoring the importance of considering species differences when translating insights from murine models to human contexts.

Few previous studies have investigated the impact of peripheral entrainment juxtaposed with intraventricular delivery on human myeloid differentiation potential. In murine studies, fetal liver monocytes have been shown to generate brain myeloid cells that fully recapitulate the microglial signature, but human myeloid inquiries into these areas are notably lacking. Peripheral immune cells may enter the CNS through the blood-brain barrier, blood-cerebrospinal fluid barrier, or subarachnoid vasculature within the leptomeninges^17,87^. Utilizing a fetal liver delivery approach, we found that diverse myeloid populations, but not primary human microglia, can migrate to and occupy the open CNS myeloid niche. iPSC derived progenitors retained aspects of microglial-like differentiation potential, both in marker expression and overall transcriptional state despite their entrainment in the periphery. The trend towards accumulation of LYVE1^+^ iPSC derived myeloid cells in perivascular regions, and a greater proportion of cells in perivascular macrophage single cell clusters suggests perivascular migration and possibly perivascular entrainment with peripheral engraftment. Surprisingly, human monocytes and CD34^+^ HSCs had a reduced capacity to differentiate to perivascular BAMs both histologically and transcriptionally, also contrary to murine studies. Notably, amongst bone marrow HSCs derived cells, peripheral engraftment led to an increased proportion of MHC and interferon populations in single cell sequencing paralleled by a tenfold increase in CD68 immunoreactivity, whereas CNS engraftment conversely led to increased cellular stress and early aging signatures. Interestingly, adult CNS xenotransplanted monocytes in the spatial transcriptomic dataset from Davtvan and Chadarevian et al. resembled peripherally delivered umbilical cord blood derived cells, perhaps reflecting myeloid entrainment. Altogether we find that ontogeny is the primary driver of brain myeloid differentiation potential, but with subtle but consistent effects of entrainment from prior environment.

Epigenetic transcription factor network modeling identified candidate transcription factors underlying and influencing these different human brain myeloid cell fates. We found the peripheral myeloid program is driven by IRF and STAT transcription factors resulting in upregulation of interferon target genes. IRF1 is critically involved in the stress responses^88^, and regulates chromatin accessibility involved in amplifying the interferon response in HSCs derived myeloid cells, but not monocytes after TLR4 stimulation^48^. IRF-responsive microglia are known to engulf entire neurons during cortical development, leading to an excitatory/inhibitory imbalance in the deeper cortical layers and promoting cortical remodeling^89,90^, a process that is potentially hyperactivated in peripheral myeloid cells. Upregulation of MITF/TFE drive autophagosome biogenesis, enhancing the phagocytic capacity^47,64,91^ of HSCs derived myeloid cells, which we find reflected in higher expression of CD68, PICALM, and SPP1^81,92–94^ and the increased phagocytic propensity of HSCs derived myeloid cells ex vivo and reduced excitatory and inhibitor synapses as well as demyelination in vivo. Hyperphagocytic microglia are frequently associated with increased production of reactive oxygen species (ROS), a process primarily mediated by the enzyme NADPH oxidase^95,96^. Our findings reveal elevated ROS levels in monocyte-derived myeloid cells, indicative of excessive inflammation and contributing to myeloid-mediated neurotoxicity^97,98^. Taken together, increased phagocytosis, ROS production, and enhanced IRF signaling can trigger neuroinflammation and neuronal death, leading to a reduction in the thickness of cortical layers 5 and 6 in peripheral myeloid engrafted brains. Hyperactivation in these pathways is coupled by a loss of homeostatic transcription factor pathways. Notably the SALL1 motif is AT rich and not represented within current databases, but MEF2 pathways as well as TGF-β responsive SMADs are core to the modeling of in vivo iPSC derived microglia and are lost in HSCs and monocyte derived cells.

Pathogenic microglial responses contribute to the mechanism and progression of numerous neurological disorders, making microglia replacement therapy a promising treatment approach^99–101^. Studies of mice indicate that embryonically derived microglia are not replaced by peripheral myeloid cells under normal conditions. Whether this is the case for humans remains to be unequivocally established. Studies of postmortem brains derived from elderly, cognitively normal individuals with clonal hematopoiesis of indeterminant potential (CHIP) indicated high levels of replacement of the myeloid compartment with peripheral myeloid cells that was associated with protection from AD^72^. Since CHIP is caused by mutations that result in a proliferative advantage, it is not clear that these findings will apply to the normal human brain. A recent study^29^ primarily focused on engraftment of peripheral mouse myeloid cells into the mouse brain also briefly explored engraftment of human myeloid progenitors. However, due to the experimental protocol, these studies only achieved engraftment efficiencies of <5% of brain myeloid cells, rather than complete engraftment as in the present studies, with extensive co-engraftment of B cells. This small set of cells enabled identification of a human peripheral myeloid signature suggesting that the adult human brain contains ~2–8% peripherally derived cells, with higher percentages of these cells significantly associated with AD pathology. Notably, this study did not observe the strong type I interferon response observed with complete engraftment, suggesting that low levels of peripheral myeloid cells in the human brain may be tolerated. Current preclinical strategies involve injecting donor HSCs or monocytes into a microglia-depleted niche created by preconditioning such as irradiation or chemotherapy with the extent of engraftment likely being low in most or all protocols^102,103^. Although replacing dysfunctional microglia with optimized donor myeloid cells has shown benefits in alleviating behavioral and cognitive impairments, challenges such as limited cell sources, safety concerns, and transplantation inefficiency remain widely recognized and discussed^104^. Most importantly, whether transplanted cells can supplant the environment dependent role of yolk sac microglia is a critical unanswered question. Studies are currently conflicting, such that some show increased myeloid infiltration in pathological conditions such as Alzheimer’s^29^, whereas others suggest that myeloid infiltration may be protective^72^. These studies intersect complex neuropathology and neurodegenerative disease with myeloid effects, whereas intersection with tangle, plaques or other pathology is not queried herein. Our data show that HSC-derived brain myeloid cells lack expression of key homeostatic transcription factors, such as MEF2C, while upregulating transcription factors like STAT1/2 or IRF that lead to interferon pathway overactivation and a diseased-state phenotype. Importantly, mechanisms that are responsible for the selective activation of these factors in peripherally derived cells are unknown. It is also unknown how peripheral myeloid interferon responses interface with adaptive immunity in the physiological brain environment, and whether these responses will be altered concurrently with yolk sac–derived microglia. Strategies to mitigate these effects could be of value in further efforts to advance HSC-based approaches to microglia replacement.

In conclusion, our study demonstrates pronounced divergence between human yolk-sac and bone marrow derived myeloid cells in the murine brain environment, with human bone marrow derived myeloid cells leading to widespread deleterious effects on the brain. Our data together with the accompanying study of Davtvan and colleagues suggests that therapeutic global brain myeloid replacement may have unforeseen negative long-term impact in humans.

## Methods

### Human brain tissue

Postnatal brain tissues were collected from two patients (a 16-year-old female and a 4-year-old male) diagnosed with focal temporal lobe refractory epilepsy who underwent epileptogenic focus resections at Rady Children’s Hospital. All the procedures were obtained with informed consent from adult patients, or by informed parental consent and assent when applicable from pediatric patients under a protocol approved by the UC San Diego and Rady Children’s Hospital Institutional Review Board (IRB 160531, IRB 171361). Final pathological diagnosis, epilepsy medications, demographics, and timing of stereo electroencephalography (SEEG) are reviewed prior to surgery. Resected temporal lobe tissue was immediately placed on ice and transferred to the laboratory for microglia isolation. Microglia were directly isolated from postnatal brain tissues by Percoll gradient selections and sorted by flow cytometry. This study was performed in accordance with ethical and legal guidelines of the University of California institutional review board.

### Human cord blood samples

Fresh human umbilical cord blood (HUCB) was collected at the University of California, San Diego, Jacobs Medical Center. Informed consent was obtained from mothers of healthy newborns prior to delivery for all procedures. The protocol was approved by the UC San Diego institutional review board (IRB 160531), with sample collection supported through the UCSD Center for Perinatal Discovery (under IRB 181917). Human cord blood samples were included if obtained from healthy, full-term deliveries with adequate volume, and excluded in cases of maternal infection, pregnancy complications, or poor sample quality. UCB samples (approximately 10 ml each) were collected by gravity flow into heparinized tubes as an anticoagulant. Fresh UCB samples were immediately sent to the laboratory on ice within hours for hemopoietic stem cells isolation.

### Murine Model

The murine model used in this study is a previously published humanized immunodeficient mice genetically deficient in resident microglia (*Csf1r*^ΔFIRE/ΔFIRE^ *CSF1*^h/h^ *Rag2*^−/−^ *Il2rg*^−/−^)^31^. Humanized immunodeficient mice were sourced from Jackson Laboratory (Strain #017708) and bred to Csf1r *fms* intronic response element (FIRE) enhancer knockout mice that were a kind gift from Drs. Clare Pridans and David Hume^30^. The animals were bred, maintained, and procedures performed at the Sanford Consortium for Regenerative Medicine. The mice were maintained with enrofloxacin in the water supply and breeder chow and were kept in a pathogen-free biomedical environment under a 12h light-dark cycle. All treatments are in accordance with University of California, San Diego research guidelines for the care and use of laboratory animals.

### Human pluripotent stem cell culture

The induced pluripotent stem cell (iPSC) line EC11^31,105^ was cultured using standard techniques. Briefly, cells were cultured in mTESR plus (Stem Cell Technologies, Vancouver, BC) and routinely passaged using Gentle Cell Dissociation Reagent (Stemcell Technologies, Vancouver, BC) onto Matrigel (1 mg/ml) coated plates. Stem cell markers were validated by immunofluorescence, normal karyotype was established by standard commercial karyotyping (WiCell Research Institute, Madison, WI) and through DNA fingerprinting (IGM Genomics at the University of California, San Diego), and mycoplasma testing was regularly performed.

### Hematopoietic progenitor cell (iHPC) differentiation from iPSCs

Hematopoietic progenitor cells (iHPCs) were generated from iPSCs as previously described with minor modifications^106^. Cells were differentiated into CD43^+^ hematopoietic progenitors using the StemCell Technologies Hematopoietic Kit (Cat #05310) as has been previously published^31,32^. Briefly, iPSCs were cultured onto 6-well plates coated with Matrigel (1 mg/ml) using ReLeSR (StemCell Technologies, Vancouver, BC) containing 10 mM ROCK inhibitor. Wells were selected based on density, targeting 100 cells per well and aggregates of 10–20 cells per well. The selected wells were transitioned to basal medium containing Supplement A (1:200) on day 1, another 1 ml per well on day 3, and to basal medium containing Supplement B (1:200) on day 5 according to cell morphology. On days 7, 9, and 11, cells were supplemented with an additional 1 ml per well of medium B. On days 13 and 15, unadhered iHPCs were carefully collected from the medium and centrifuged at 300 g for 5 min. For in vitro microglia differentiation, iHPCs were grown at a density of 500,000 cells/well on Matrigel-coated plates and placed in microglia completion medium. For xenograft studies, iHPCs were collected and frozen in serum-free medium at a density of 1M and then thawed one day before xenografting.

### Isolation of primary CD34^+^ cells and monocytes from human umbilical cord blood (HUCB)

Each HUCB sample (~10 mL) was transferred to a 15 mL tube for immediate processing. Peripheral blood mononuclear cells (PBMCs) were isolated by centrifugation at 1400 rpm for 10 minutes at room temperature (acceleration/deceleration: 5/2). Red blood cells were lysed with eBioscience^™^ 1X RBC Lysis Buffer (Thermo Fisher, Cat# 00–4333-57) for 10 minutes at room temperature. PBMCs were collected by centrifugation at 1400 rpm (acceleration/deceleration: 5/5), resuspended in MACS^®^ Running Buffer (Miltenyi Biotec, 130–091-221), and counted. Monocytes were isolated from the PBMCs fraction by negative selection using the Pan Monocyte Isolation Kit (Miltenyi Biotec, 130–096-537) according to the manufacturer’s instructions. CD34^+^ cells were further purified using the CD34 MicroBeads Kit (Miltenyi Biotec, 130–046-702) following a 30-minute incubation at 4 °C. Isolated monocytes and CD34^+^ cells were validated by flow cytometry using a BD Fortessa X20 cytometer. Both monocytes and CD34^+^ cells were counted by Trypan Blue exclusion and cryopreserved in Bambanker solution (Thermo Fisher, Cat# NC9582225) for subsequent in vivo or in vitro experiments.

### Early Postnatal Transplantation myeloid cells in the CNS and peripherally

Postnatal human microglia, iPSC derived iHPCs as well as HUBC derived CD34^+^ cells and monocytes were thawed and viable cell numbers determined with trypan blue to target 300,000 cells which were then suspended in sterile 1X DPBS. Postnatal day 2 to 4 hFIRE mice (Csf1r^ΔFIRE/ΔFIRE^ CSF1^h/h^ Rag2^−/−^ Il2rg^−/−^) pups were selected for xenotransplantation if they were pink and milk-fed. CNS transplants were performed according to the method of Hasselmann et al^32^ with minor modifications. The pups were placed on ice for 2–3 minutes to induce hypothermic anesthesia. Cells were transplanted at four injection sites in the cerebral cortex using a 30-gauge needle attached to a 10 ul Hamilton syringe. For peripheral transplantation, the same cell counts of postnatal human microglia, iHPCs, CD34^+^ cells, and monocytes were transplanted intrahepatically in the pups using a 30ul total volume insulin syringe^107^. After transplantation, the pups were placed on a heating pad with nests from the mother’s cage to maintain the mother’s odor. After awakening, the pups were returned to their original cages and weaned at P21-P28. All mice were adults between 8–12 weeks of age at analysis.

### Xenotransplant human myeloid cells isolation

Brain myeloid cells from all transplants were isolated as we have previously described in Han et al^31^. The brain was manually dissected into small 2–3 mm pieces and immersed in homogenization buffer consisting of HBSS (Life Technologies, 14175–095), 1% bovine serum albumin (Sigma-Aldrich, A3059) and 1mM EDTA) for mechanical dissociation using a 2 ml polytetrafluoroethylene pestle (Wheaton, 358026). Brain homogenate was pelleted, filtered through a 70mm and 40mm filter respectively, re-suspended in 37% isotonic Percoll (Sigma, P4937) and centrifuged at 600×g for 30 min at 16–18°C with no acceleration and deceleration. Following Percoll gradient centrifugation, pelleted cells were collected and washed twice with homogenization buffer, filtered with a 40 mm strainer (BD Falcon 352350) and incubated with Fc-receptor blocking antibody (Human TruStain FcX, BioLegend 422302) in homogenization buffer for 15 minutes on ice. Then cells were stained with the following cell surface marker antibodies for 30 min on ice (1:100 dilution, all from BioLegend): CD11b-PE (301306), CD45-APC/Cy7 (304014), CD64-APC (305014), CX3CR1-PerCP/Cy5.5 (341614), CD14-AF 488 (301811), HLA-DR-PE/Cy7 (307616). CD11b and CD45 were included to characterize myeloid cells as a gate for sorting xenotransplant myeloid cells. DAPI (Thermofisher, 62248) was added to the samples for viability discrimination immediately prior to sorting (1 mg/ml final concentration). Microglia were purified with BD FACS Aria II defined as live/DAPI- CD11b^+^CD45^+^ single cells. Flow cytometry data were analyzed using FlowJo software (10.8.1, BD).

### Tissue processing for immunostaining

Mice were euthanized by CO2 asphyxiation and intravascularly perfused with 100mL of phosphate buffered saline (PBS) and the brain carefully dissected^108^. For fixation, brain tissue was fixed in 4% formaldehyde in PBS for one day then transferred to 30% sucrose. The tissue was sectioned in 30-um sections using a cryostat (Leica, CM1860UV). Sections were stored in tissue cryoprotectant buffer (a mixture of 250 mL glycerin and 300 mL ethylene glycol, brought up to 1000 mL with pH 7.4 phosphate buffer) at 4°C until staining.

### Immunofluorescence staining

For immunofluorescence, each brain section was plated in a well of a 24-well plate and rinsed in PBS 3 times, then permeabilized and blocked for non-specific binding in blocking buffer containing 1% Bovine serum albumin (BSA) and 0.1% Triton X-100 (Sigma X100) in a humidified chamber for 1hr at room temperature. Slides were then incubated with the appropriate primary antibodies diluted in blocking buffer at 4°C overnight. Primary antibodies include in the **Supplementary Table 3**. The next day, sections were washed twice (10 minutes each) in PBS, and incubated with fluorophore-conjugated secondary antibodies diluted in PBS solution at RT for 2 hrs. After the two-hour incubation, sections were counter stained with DAPI for 10 minutes, rinsed three times in PBS (10 minutes each), and mounted with Shandon Immuno-Mount (Thermo Fisher Scientific, 9990412). Imaging was performed on an Andor Dragonfly 200 Spinning Disk confocal microscope or Keyence Fluorescence microscope Bz-X1000 Series.

### Flow cytometry

Xenotransplanted myeloid cells were identified and characterized by a flow cytometric multicolor panel. Isolated myeloid cells were incubated with Fc-receptor blocking antibody (Human TruStain FcX, BioLegend 422302) in homogenization buffer (5%BSA with 0.5M EDTA in HBSS buffer) for 15 minutes on ice. A multicolor panel of cell surface marker antibodies (1:100 dilution, all from BioLegend: CD11b-PE (301306), CD45-APC/Cy7 (304014), CD64-APC (305014), CX3CR1-PerCP/Cy5.5 (341614), CD14-AF 488 (301811), HLA-DR-PE/Cy7 (307616) was used for staining on ice for 30 min. DAPI (Thermofisher,62248) was added to the samples for viability discrimination (1 mg/ml final concentration). Single control, isotype and negative controls were prepared for reference. Data were acquired by BD FACS Aria II sorter and analyzed by FlowJo software (10.8.1, BD). Forward and side scattering parameters were used to exclude debris, DAPI exclusion to isolate live cells.

### Brain myeloid mRNA isolation

Xenotransplanted brain myeloid cell pellets were collected after sorting and centrifuged at 500g for 5min. The pellets were dissolved in TRIzol LS (Life Technologies)and were stored at −80 for mRNA isolation. mRNA was separated by adding the same volume of chloroform and precipitated by isopropanol. The pellets were labelled by Glycoblue and washed with 80% ethanol. The concentration of mRNA was quantified by Qubit assay and lysate in RNase-free water for further library construction.

### Bulk RNA-seq library construction and sequencing

Bulk RNA-seq libraries were prepared as previously described^19^. Briefly, 100ng mRNA from brain myeloid cells were bound with Oligo d(T) Magnetic Beads (New England BioLabs S1419) and fragmented in 2x Superscript III first-strand buffer (ThermoFisher Scientific 18080051) with 10mM DTT (ThermoFisher Scientific 18080044) at 94 °C for 9 minutes. Fragment mRNA was used for synthesizing first-strand cDNA by incubating with Random primers (3 ug/ul) (ThermoFisher Scientific 48190011), 50uM Oligo dT primer, (ThermoFisher Scientific, 18418020), 20U/ul SUPERase-In (ThermoFisher Scientific AM2694), 10mM dNTPs at 50 °C for one minute and incubated with 10mM DTT, 1%Tween-20 (Sigma), Actinomycin D (2 mg/mL), and Superscript III (ThermoFisher Scientific) at 25 °C for 10min, then 50 °C for 50 min. Second-strand cDNA was then generated with dUTP at 16 °C for 2h. The dsDNA underwent end repair, dsA-tailing and adapter ligation. After each step, the dsDNA product was purified by resuspension in 20% PEG8000/2.5 M NaCl using SpeedBeads (Thermo Fisher Scientific 651520505025). Based on the qPCR results, 12–15 cycles of final PCR were performed, and the 200–500 bp DNA product was screened by gel extraction and purified using Zymo DNA Clean & Concentrator columns according to the instructions. All dsDNA samples were mixed as libraries and sequenced on an Illumina NovaSeq X Plus 10B.

### Single-cell RNA-seq

Single-cell RNA-seq was performed as previously described^31^. Sorted brain myeloid cells were centrifuged at 500g for 5 minutes and the pellets was utilized after carefully aspirating supernatant. An aliquot of the cell suspension was mixed with Trypan Blue to quantitate viability using a hemocytometer. Cells were diluted to 10k/ul resuspending in PBS. 10,000 cells (viability 65–100%) were loaded onto a Chromium CHIP (10x Genomics). Libraries were generated according to manufacturer specifications (Chromium Single Cell 3’ Library and Gel Bead Kit v3, 1000075; Chromium Single Cell 3’ Library Construction Kit v3, 100078; Chromium Chip B Single Cell Kit, 1000153; Single Index Kit T Set A, 1000213). cDNA was amplified for 16 PCR cycles. SPRISelect reagent (Beckman Coulter B23318) was used for size selection and clean-up steps. Final library concentration was assessed by Qubit dsDNA HS Assay Kit (Thermo Fisher Scientific Q32851) and fragment size was checked using the High Sensitivity D1000 ScreenTape assay on a Tapestation 4200 (Agilent). Libraries were sequenced using a NovaSeq S4 or NovaSeq X Plus 10B (Illumina).

### ATAC-seq library preparation

ATAC-seq libraries were prepared as previously described^109^ using approximately 50,000 sorted brain myeloid cells. Cell pellets were resuspended in 50 μl of ice-cold lysis buffer (10 mM Tris-HCl, pH 7.5, 10 mM NaCl, 3 mM MgCl2, and 0.1% IGEPAL CA-630) and centrifuged at 500 g for 10 minutes. The lysed pellets were incubated in 20 μl of the transposase reaction mix (10 μl ATAC Tagment DNA buffer, 1 μl Tagment DNA enzyme I) at 37°C for 30 minutes. DNA was purified using the Zymo Research ChIP DNA Clean & Concentrator Kit (Zymo Research D5205) and eluted in 11 μl of elution buffer. PCR amplification was performed using the NEBNext High-Fidelity PCR Master Mix with 10 μM Nextera Primer Ad1 and a unique barcoding primer Ad2 for 10 cycles. The library was assessed on a 10% TBE gel, run at 70 V for 30 minutes followed by 140 V for 1 hour. The gel was stained with SYBR Gold (diluted 1:10,000) and the fragment size between 175–225 bp was excised. Gel slices were transferred to 0.5 ml low-binding tubes, centrifuged at maximum speed for 2 minutes at room temperature, and incubated with diffusion buffer for 1 hour at room temperature. DNA was then isolated using the Zymo Clean & Concentrator Kit and eluted in 12 μl of elution buffer. The DNA concentration was measured using Qubit and stored at −20°C prior to sequencing.

### ChIP-seq library preparation

Chromatin immunoprecipitation (ChIP) was performed in biological replicates as previously described^110^, with the following modifications. Brain myeloid cells were isolated and stained with CD45 and CD11b antibodies. For H3K27ac ChIP assays, labeled cells were crosslinked with 1% (v/v) formaldehyde in PBS for 10 minutes at room temperature. Crosslinking was quenched by adding 2.5 M glycine and incubating for 5 minutes at room temperature. CD45^+^CD11b^+^ cells were subsequently sorted, and cell pellets were obtained by centrifugation at 1600 × g for 5 minutes at 4 °C. Crosslinked cells were resuspended in ice-cold LB3 buffer (10 mM Tris-HCl, pH 7.5; 100 mM NaCl; 1 mM EDTA; 0.5 mM EGTA; 0.1% sodium deoxycholate; 0.5% N-lauroylsarcosine; 1 mM PMSF; 0.5 mM sodium butyrate; and 1× protease inhibitor cocktail). Chromatin was sheared using a Covaris E220 sonicator in microTUBE AFA Fiber pre-slit snap-cap tubes (Covaris) for 12 cycles under the following settings: 60 seconds per cycle, 5.0% duty factor, and 140 peak incident power (PIP). After sonication, lysates were diluted with 10% Triton X-100. A 1% aliquot of the lysate was reserved as input control. The remaining lysate was incubated overnight at 4 °C with specific antibodies pre-bound to a 1:1 mixture of Dynabeads Protein A and Protein G (10 μl each, total 20 μl per reaction). Beads were collected using a magnetic stand and washed three times with washing buffer supplemented with 1 mM PMSF, 0.5 mM sodium butyrate, and 1× protease inhibitor cocktail. ChIP-enriched chromatin was used for library preparation with the NEBNext Ultra II DNA Library Prep Kit (New England Biolabs) following the manufacturer’s instructions. To reverse crosslink, 46.5 μl of eluate was combined with 4 μl of 10% SDS, 4.5 μl of 5 M NaCl, 3 μl of 500 mM EDTA, 1 μl of 20 mg/mL Proteinase K, and 20 μl of water. Samples were incubated for 1 hour at 55 °C followed by 30 minutes at 65 °C. DNA was purified using 2 μl of SpeedBeads in 20% PEG 8000/1.5 M NaCl and eluted in 25 μl of TT buffer (10 mM Tris-HCl, 1 mM EDTA, 0.2% Tween-20, 1 mM PMSF, 0.5 mM sodium butyrate, 1× protease inhibitor cocktail). Libraries were size-selected for 225–325 bp fragments using PAGE/TBE gel electrophoresis followed by gel extraction. Final DNA was purified using the ChIP DNA Clean & Concentrator Kit (Zymo Research) and sequenced on the Illumina NovaSeq X Plus platform (10B flow cell).

### RNAscope

RNAscope probes targeting *APOE* and *MS4A7* were obtained from Advanced Cell Diagnostics (ACD). Tissue slides were frozen and dried at −20°C for 2 hours, and the protocol outlined in the RNAscope Multiplex Fluorescent Reagent Kit V2 User Manual was followed. Briefly, sections were washed in PBS for 5 minutes and baked at 60°C for 30 minutes. Samples were fixed in 4% PFA for 15 minutes at 4°C, followed by dehydration through a graded ethanol series (50%, 70%, and 100%) for 5 minutes each. Following dehydration, sections were incubated with ACD H_2_O_2_ for 10 minutes, rinsed in water, and treated with target retrieval buffer (ACD) at 98–100°C for 15 minutes. Sections were washed in water, treated with 100% ethanol, and then incubated with Protease III at 40°C for 30 minutes in the RNA scope HybEZ oven. Hybridization of probes was performed by adding 2 drops per section, followed by incubation for 2 hours at 40°C. Post-hybridization washes were conducted using ACD wash buffer. Amplification was carried out sequentially: Amp 1 (30 minutes at 40°C), Amp 2 (30 minutes at 40°C), and Amp 3 (15 minutes at 40°C), with each step followed by two 2-minute washes in wash buffer. For signal detection, HRP-C1 was added for 15 minutes at 40°C, followed by washes in buffer. C1 probe detection (1:1000 dilution, TSA Vivid Fluorophore) was performed for 30 minutes at 40°C, followed by two washes and blocking with RNAscope Multiplex FL v2 HRP blocker for 15 minutes at 40°C. The sections were subsequently immunostained for IBA1 by incubating overnight at 4°C with a primary antibody diluted in 10% BSA and 1% Triton X-100. Sections were washed three times for 10 minutes each in PBS, incubated with a secondary antibody (1:1000 dilution) for 2 hours at room temperature, and washed twice for 10 minutes in PBS. DAPI (1:1000 dilution) was applied for 10 minutes, and slides were mounted using Immuno-Mount. Slides were allowed to dry overnight at room temperature in the dark. Images were captured using a Dragonfly confocal microscope with a 63x objective lens. Image processing and analysis were performed using Fiji (v2.14.0).

### Imaging analysis

Immunofluorescence images were analyzed using Fiji (v2.14.0)^111^, CellProfiler (v4.2.8)^112^, or Imaris (v10.1.0)^113,114^. Microglial and astrocyte morphology was assessed in CellProfiler by quantifying branches, endpoints, signal intensity, and area from skeletonized IBA1^+^ or Gfap^+^ structures. Primary objects were segmented using an adaptive Otsu threshold (10–50 pixel size range) based on Ku80 or Sox9-stained nuclei. The *MeasureObjectSkeleton* module in CellProfiler was used to quantify skeletonized branches and endpoints as measures of ramification complexity. Expression of microglial markers (P2RY12, MX1, HLA-DR, CD9, PLIN2, as well as transcription factor MEF2C and phosphor-STAT1) and RNAscope signals was quantified in Fiji. Z-stacks were split into individual channels, and brightness/contrast were standardized. IBA1^+^ cells were identified, and ROIs were used to quantify marker intensity, area, and percent positivity. Myelin sheath morphology was analyzed in Imaris using Gaussian filtering, background subtraction, and machine learning-based surface segmentation of Mbp^+^ structures (>100 surfaces). For synaptic analysis, 3D volumes were cropped (x: 300 μm, y: 300 μm, z: 1–5 μm), and surfaces were generated with a 0.5 μm seed diameter. Postsynaptic binding sites were manually quantified in 2D/3D, and binding efficiency was calculated as the percentage of synapses with colocalization. Five cropped regions per image were analyzed across three cortical areas, using samples from three animals per group.

### Western blot analysis

Whole brains or cortical regions were harvested from xenotransplanted mice 8 weeks post-transplantation. Brain tissues were homogenized in ice-cold lysis buffer (1× RIPA buffer supplemented with a protease inhibitor cocktail; Fisher Scientific Cat# 78443) and incubated on ice for 10 minutes. Lysates were centrifuged at 21,000 × g for 10 minutes at 4°C, and the resulting supernatants were collected. Protein concentrations were quantified using the BCA Protein Assay Kit according to the manufacturer’s instructions. Equal amounts of protein (40 μg) were separated on NuPAGE 4–12% Bis-Tris gels (Novex) and transferred onto PVDF membranes using standard wet transfer protocols. Membranes were blocked and incubated overnight at 4°C with the following primary antibodies: anti-IFITM3 (Proteintech, Cat# 11714–1-AP), anti-myelin basic protein (Millipore Sigma, Cat#AB9348), anti-vGlut2 (Cell Signaling, Cat#71555), anti-Gad67(Thermo fisher, Cat#PA5–21397), anti-GAPDH (Santa Cruz, Cat# sc-47724) and anti-β-actin (Cell Signaling, Cat# 3700). GAPDH and β-actin served as internal loading controls. Following primary incubation, membranes were washed and incubated with IRDye-conjugated secondary antibodies: anti-rabbit IgG (800CW) and anti-mouse IgG (680RD) (LICOR Biosciences, Lincoln, NE, USA). Protein bands were visualized using the Odyssey Infrared Imaging System (LI-COR Biosciences), and band intensities were quantified using Image Studio Lite 4.0 software.

### Ex vivo phagocytosis and reactive oxygen species (ROS)

Brain myeloid cells were isolated from brain as described above using Percoll gradient centrifugation. Briefly, synapses were isolated using the Syn-PER synaptic protein extraction reagent (Thermo Fisher Scientific, Cat#87793) per the manufacturer’s instricuctions. Myelin was collected from brain tissue by manual dissociation of the myelin layer following Percoll gradient centrifugation. The isolated synapses and myelin were labeled with pHrodo^™^ iFL Red Microscale Protein Labeling Kit (Thermo Fisher Scientific, Cat#P36014) for 1h at RT following the manufacturer’s instructions. **Purified tau proteins were labeled with pHrodo**^**™**^ **Red dye**, and **Aβ proteins were labeled with NHS Ester-conjugated Alexa Fluor**^**™**^ **488 dye** (Thermo Fisher Scientific, Cat# A20000) according to the manufacturer’s instructions. For phagocytosis assessment, xMGs were incubated with **pHrodo-labeled synapses, myelin, Human recombinant Tau-441 protein pre-formed fibrils (Acro systems, TAU-H5115), and Fluor 488 labeled Aβ** (amyloid-beta 1–42) (Anaspec, AS-60479–01) for 1 hour at 37°C. Following incubation, brain myeloid cells were washed thoroughly with 1% bovine serum albumin (BSA) in PBS buffer to remove unbound material. To measure ROS production, brain myeloid cells were incubated with **CellROX** (Thermo Fisher Scientific, Cat#C10443) for 30 minutes at 37°C, according to the manufacturer’s protocol. After ROS labeling, the cells were washed with 1% BSA in PBS. For surface marker staining, the cells were further incubated with **FITC-conjugated anti-CD11b** antibody (BioLegend, Cat# 301330) or PE anti-CD11b ((BioLegend, Cat# 301306) for 25 minutes on ice to identify microglial populations. After staining, the cells were washed, and double-positive populations (e.g., FITC-CD11b with pHrodo-labeled or CellROX-labeled signals) were analyzed by **flow cytometry** (BD LSRFortessa^™^ X-20 Cell Analyzer). Data acquisition and analysis were performed using **FlowJo software** (10.8.1, BD). All experiments were conducted under standardized conditions, and appropriate controls were included to validate phagocytosis and ROS measurement.

### Cytokine measurement

Brain lysates were prepared as described above and analyzed for cytokine expression using the Human Cytokine Array C5–8 Kit (RayBiotech, Cat# AAH-CYT-5–8), according to the manufacturer’s instructions. Briefly, lysates were incubated with the cytokine array membranes, which are pre-coated with specific antibodies for detecting multiple human cytokines. The membranes were washed to remove unbound proteins and incubated with biotin-conjugated antibodies, followed by a streptavidin-HRP incubation step. Chemiluminescent detection reagents were applied, and the signal was visualized using a ChemiDoc Bio-Rad imaging system. Spot intensities corresponding to individual cytokines were quantified using densitometry with Fiji software. Background signal was subtracted, and data were normalized to internal positive controls on the array membrane.

### RNA-seq and ATAC-seq preprocessing and mapping

RNA-seq FASTQ files were processed and aligned to the hg38 genome using STAR^115^ with default parameters. For ATAC-seq data, reads were trimmed to 30 bp and subsequently aligned using Bowtie2 (v2.3.5.1)^116^. Following alignment, HOMER (v4.10)^117^ was employed to process the mapped reads, converting them into “tag directories” using the makeTagDirectory command. HOMER was also utilized to generate sequencing-depth normalized bedGraph and bigWig files, which were visualized using the UCSC Genome Browser (http://genome.ucsc.edu).

### Bulk RNA-sequencing analysis

FASTQ-files were mapped to the UCSC genome build hg38 with STAR (v2.5.3a) using default parameters and converted to HOMER tag directories. The function “analyzeRepeats” was used to quantify raw reads and then normalize reads as either counts per million (CPM) values or transcripts per million (TPM). A pseudocount of 1 TPM/CPM was added to each gene before base-2 logarithm transformation of TPM/CPM for each gene. Differential expression was performed from counts using R (v4.3.1) and the package DESeq2 (v1.44.0) with an FDR<0.05 and log2 Fold Change > 1 on 4–7 biological replicates. Postnatal microglia TPM and counts were previously published^31^. Batch correction was used with CombatSeq as previously described, for PCA visualization^118^. Additional covariates included in the DESeq2 design were batch, cell type, environment and species depending on the pairwise comparison or one-way ANOVA required. Pathway enrichment was performed using Metascape^119^. Additionally, Ingenuity Pathway Analysis software (Qiagen, Germantown, Maryland) was utilized to perform both canonical pathway enrichment and upstream regulator analysis^120^. Analysis of patterns of gene expression were performed the degPattern function of the package DEGreport (v1.44.0). Clusters of genes are represented as z-scores of abundance. Volcano Plots for pairwise comparisons are generated using ggplot2 (v3.5.1). Heatmaps for pairwise and one-way ANOVA comparisons across multiple groups comparisons were generated using log2(TPM+1) data using Pheatmap (v1.0.12).

To determine the overall concordance between datasets (ours and Davtvan and Chadarevian et al), we calculated the differential expression outputs of both Visium pseudobulking and RNA-seq comparing microglia- and monocyte-specific gene expression. Unlike conventional overlap analyses that rely on applying an arbitrary log2 fold-change cutoff to define differentially expressed genes, we instead ranked all genes in each dataset based on log2 fold change and statistical significance (padj < 0.05). These genome-wide ranked lists were then compared using the Rank-Rank Hypergeometric Overlap (RRHO) method, which evaluates overlap across the entire distribution of ranked genes rather than restricting analysis to a pre-defined threshold. The ranked lists were then compared using the RRHO method to identify regions of significant overlap between up- and downregulated genes, with the significance of overlaps evaluated by hypergeometric testing, as previously described^121^. Co-regulated genes are genes that are ranked similarly between datasets based on fold change and statistical significance criteria. Pathway enrichment of co-regulated genes was performed using Metascape^119^.

### Mouse/Human Comparison

External datasets used for species comparison of monocytes (MNC) and CD34 cells included: Bennett et al., 2018^27^ and Cronk et al., 2018^23^. The Bennett et al., 2018^27^ dataset included the following mouse cell types: (1) primary microglia (WT MG); (2) xenotransplantation of microglia (ICT MG); (3) HSCs-derived cells from blood (ICT MNC), (4) HSCs-derived cells from bone marrow (ICT CD34). For Bennett et al., 2018, raw fastq files were attained from the National Center for Biotechnology Information (NCBI) Bioproject, accession no. PRJNA453419. Samples were processed as described above. Batch between species and experiments was corrected using CombatSeq as previously described, for PCA visualization^118^. First, to ensure that there was no difference between mouse CD34 and mouse Monocytes (MNC), we performed a pairwise differential expression analysis. Since only 11 genes were differentially expressed (padj<0.05), we chose to pool these downstream to create a mouse CD34/MNC category for comparisons with human primary microglia and human CD34/MNC pooled data. For these comparisons, a pairwise differential expression approach using DESeq2 was performed, correcting for cell type, species and batch in the design. Significance of overlaps between differentially expressed genes and categories was done using a hypergeometric p-value. Pathways enriched in murine-specific, shared and human-specific categories was performed using Metascape^119^. IPA was used to determine upstream regulators for in murine-specific, shared and human-specific categories. Heatmaps for comparisons were generated using log2(TPM+1) data using Complex Heatmap (v 2.16.0)^122^.

To determine the degree of similarity of our dataset, including mouse and human samples, to brain engrafting macrophages, we leveraged the data reported in Cronk et al., 2018^23^. For Cronk et al., to isolate the brain-engrafting macrophages (beMφs) specific signature, the top differentially expressed genes (DEGs, padj<0.05) were taken from each reported model (Genetic model, Traditional BMT, BMT/PLX5622) then overlapped to find ~50 overlapping genes, defined as the beMφ pooled DEG signature. The data for the beMφ pooled DEG signatures was first taken as a DESEq2 dds object per species, then rlog transformation of the dds object, followed by z-score scaling. Complex Heatmap (v.2.16.0) produced the final heatmap shown in **Extended Data Fig. 4f** comparing human and mouse CD34/MNC and microglia to this brain-engrafting macrophage signature.

### Single cell RNA-seq analysis

Raw sequencing data was demultiplexed and preprocessed using the Cell Ranger software package (v7.2) (10x Genomics). Raw sequencing files were first converted from Illumina BCL files to FASTQ files using cellranger mkfastq for scRNA-seq. Demultiplexed FASTQs were aligned to the GRCh38 reference genome (10x Genomics) and reads for exonic reads mapping to protein coding genes, long non-coding RNA, antisense RNA, and pseudogenes were used to generate a counts matrix using cellranger count; expect-cells parameter was set to 5,000. Fastq files were processed using Cell Ranger (v.7.2) software to process barcodes and demultiplex reads using default parameters. The resulting count matrices were filtered and analyzed using Seurat package (v.4.4.0).

Cells that had fewer or greater than 200 and 7000 features, respectively, and contained greater than 5% of reads from mitochondrial genes and 6% of reads from ribosomal genes were considered low quality and removed from further analysis. No software (such as DoubletFinder) was used to infer and remove doublets owing to the risk of accidentally removing transitioning cell substates. Instead, we manually examined the data for unexpected co-localization of well-known cell-type-specific gene markers. Data were normalized and scaled using the Scale Data function. Data were integrated based on 2000 highly variable genes using the FindVariableFeatures() function. Principal component analysis and UMAP dimension reduction were performed using the RunPCA() and RunUMAP() functions (dims = 1:10). A nearest-neighbor graph was then calculated using the FindNeighbors() function (dims = 1:10), followed by clustering using the FindClusters() function (resolution = 0.3). Clustree (v0.5.1) was used to assist in visualization of appropriate resolution.

Harmony (v1.2.3) was used to remove batch effects across samples as previously described^123^. Integration across batch was evaluated using PCA, harmony embeddings and UMAP by batch, group and condition. Cluster identity was determined by finding differentially expressed genes for each cluster using the FindMarkers() function and comparing the identified markers to known cell-type-specific genes. Clusters were determined using gene input lists and the package scSorter (v0.0.2) for semi-supervised cluster assignment, as previously described^124^. Reference datasets for microglia subsets include Homeostatic^19^; Antigen presenting microglia (MHC)^53^; Lipid droplet accumulating microglia (LDAM)^53^; Interferon-responsive microglia^52^; Cycling microglia^56^; Neuronal surveillance microglia^56^; Proliferative-region-associated microglia (PAM)^51^; Disease associated microglia (DAM)^32,52^; perivascular macrophages^55^; and aging^54^. Enrichment of the Visium spatial DEG2 signature from Davtyan and Chadarevian et al. was assessed using Seurat’s AddModuleScore function and visualized on UMAPs. FeaturePlot() and Seurat was used to show average expression of specific microglia signatures across clusters. Specific genes are plotted using VlnPlot() as a function of expression level across clusters.

### ATAC-seq analysis

Peaks were called using HOMER’s findPeaks command with the following parameters: “-style factor -size 200 - minDist 200” from tag directories for ATAC-seq experiments. After merging peaks using HOMER’s mergePeaks tool, replicate consistency within each cell subset or engraftment region was assessed by calculating pairwise correlations based on tag counts. The two replicates exhibiting the highest correlation were selected for peak reproducibility analysis using the Irreproducible Discovery Rate (IDR) framework. Peaks with an IDR < 0.05 were retained for downstream analyses to ensure high-confidence peak selection. Peaks were annotated using HOMER’s annotatePeaks.pl using all tag directories. Subsequently, DESeq2 was used for pairwise comparisons to identify the differentially chromatin accessible distal sites (1000bp away from known TSS) or proximal sites (<500bp away from known transcript) with p-adj < 0.05 and fold change > 2. De novo motif analysis was performed using HOMER’s findMotifsGenome.pl with either nonsignificant peaks or random genome sequences generated by HOMER as background. Motif enrichment scoring was performed using binomial distribution under HOMER’s framework. Motif enrichment was visualized using ggplot2 to form bubble plots with color as a function of − log10P enrichment and size a function of target percentage (%).

### ChIP-seq analysis

FASTQ files were filtered to retain reads trimmed to 50 bp and then aligned to the human reference genome (hg38) using Bowtie2. Tag directories for each group were created and merged using the makeTagDirectory command in HOMER. Peak tracks were visualized in the UCSC Genome Browser using HOMER’s makeMultiWigHub.pl utility. To quantify differential H3K27ac enrichment between genotypes (CNS_iHPCs vs. CNS_CD34/monocytes_combined; Peripheral_iHPCs vs. Peirpheral_CD34/monocytes_combined), IDR-filtered ATAC-seq peaks from the respective comparison groups (CNS and periphery) were merged using the mergePeaks function in HOMER. The resulting merged peak set was annotated with trimmed H3K27ac tag counts from each sample using annotatePeaks.pl with a window size of 1000 bp. Peaks with low signal (tag count <4) were removed using the filter_low_tag_counts.pl script. Differential H3K27ac signal was then identified using HOMER’s getDiffExpression.pl function, applying a fold-change cutoff of >2 and an adjusted p-value threshold of <0.05. Motif enrichment analysis was performed using HOMER’s findMotifsGenome.pl , incorporating both known and de novo motifs with motif lengths of 8, 10, 12, 14, and 16 bp, maximum mismatches 4. Background peaks were generated automatically by HOMER using random genomic sequences (GC background).

### TIMON analysis

We applied our published innovative computational framework, TIMON^31^, to identify significantly enriched co-occurring transcription factor (TF) motifs, enabling the elucidation of regulatory dynamics underlying human myeloid cell ontogeny. In brief, TIMON identifies over-represented motif pairs from active chromatin elements (open chromatin, enhancer etc.) and construct TF co-occurring networks to infer TF coopertivities. For our analysis, we applied TIMON to differential H3K27ac peaks overlapping ATAC-seq regions (hereafter referred to as active enhancers) derived from upregulated CNS-iHPCs and CNS-CD34/monocytes_combined samples as input. Enhancers were identified following an approach similar to our previously published work^125^. A list of differentially expressed TFs, derived from RNA-seq data, was used to compute co-occurrence frequencies of non-overlapping motifs. Statistically significant TF motif pairs (edges) were identified using a p-value threshold of < 0.001 and visualized as a motif interaction network. Maximal cliques within the network were extracted to quantify motif frequency. Hub motifs and unique network edges were identified to assess regulatory specificity. Motif enrichment analysis was performed using **HOMER** ( findMotifsGenome.pl ) with a 200 bp window and HOMER’s internal motif database. Shared TFs across overlapping peak regions were identified, and their target genes were annotated using annotatePeaks.pl . Gene Ontology (GO) enrichment analysis was then conducted to identify biological pathways associated with common TF targets. Finally, representative genes within differential peaks were visualized using the UCSC Genome Browser to highlight region-specific regulatory patterns.

### Linkage disequilibrium score regression

Linkage disequilibrium score regression (LDSR) was used to estimate the heritability enrichment of neurodevelopmental, neurodegenerative, neuropsychiatric and autoimmune traits at genomic regions of interest. Specifically, regions with significant active enhancer elements defined by histone modifications at accessible chromatin regions in both CNS CD34^+^/monocytes and CNS iHPSs were selected. Using the coordinates of these intersecting regions, SNP lists were compiled from all annotated variants within a 5kb window around the centers of these regions. LD scores for the identified SNPs were calculated using European ancestry reference data from HapMap3 and 1000 Genomes Phase 3, using the approach outlined by the pervious study^126,127^. Summary statistics from relevant GWAS studies were curated from the NHGRI-EBI catalogue^128–137^. To account for differences in file formatting and ensure harmonization, the Bioconductor based *MungeSumstats* package was used to process data from each study^138^. The Bulik-Sullivan baseline LDSC model was used to regress GWAS test statistics against the LD scores of the annotated SNPs to obtain heritability estimates^139^. This approach then allowed for the calculation of heritability enrichment by comparing the proportion of total heritability attributable to SNPs within the defined regions to their relative proportion of total heritability captured across the genome for that trait.

### Statistical analysis

Gene expression differences were analyzed using the **DESeq2** package, applying the Benjamini-Hochberg multiple testing correction to control for false discovery rate. Genes were considered differentially expressed if they met the thresholds of log2 fold change (FC) > 1 and adjusted p-value (p.adj) < 0.05. For comparisons between experimental groups, statistical significance was assessed using one-way analysis of variance (ANOVA), followed by post-hoc multiple comparison corrections (Tukey’s test). Significance levels are reported as follows: *p* < 0.05 (*), *p* < 0.01 (**), *p* < 0.001 (***), *p* < 0.0001 (****), *n.s.* indicates non-significant differences. Data are presented as mean ± standard error of the mean (SEM) or standard deviation (SD), as specified in the figure legends. All analyses were performed using R or GraphPad Prism software.

## Supplementary Material

Supplementary Files

This is a list of supplementary files associated with this preprint. Click to download.

• SupplementaryTable1Ontogenyandenvironmentdrivetranscriptioninbrainmyeloidcells.RelatedtoFigure2andExtendedDataFigure2.xlsx

• SupplementaryTable2Speciescomparisonofhumanmyeloidsignatures.RelatedtoFigure4andExtendedDataFigure4.xlsx

• SupplementaryTable3Keyresourcesusedforimmunofluorescencecellculturelibrarypreparationandbioinformaticanalysis.xlsx

• ExtendDataFigures.pdf

• ExtendedDataFigureLegends.docx

## Data Availability

Previously reported data are available from GEO including Gosselin et al.^19^: GSE62826, Han et al.^31^: GSE226690. Primary human epigenetic data is available on dbGAP for Nott et al.^35^ on dbGAP: phs001373.v2.p2. Code for TIMON analysis is previously published^31^ and available here; https://github.com/rzzli/FetalMicroglia. Murine brain myeloid data is previously published and available at NCBI under Bioproject accession number PRJNA453419^27^ and GEO GSE84819^23^. Data generated from this study are accessible under the SuperSeries GSE307264. All code used for the analysis of RNA-seq, ATAC-seq, and H3K27ac ChIP-seq data in this manuscript is available through HOMER (http://homer.ucsd.edu/homer/) and on Coufal lab GitHub (https://github.com/Coufal-Lab/MicrogliaTools)
